# Spirit of the Foreign Periodicals, &c.

**Published:** 1839-01-01

**Authors:** 


### Spirit of the Foreign Periodicals, &c.

### Discussion at the Royal Academy on the Contagiousness of Plague and other Fevers.

M. Renoult introduced the subject by reading some observations on the Plague, which he had collected together during his residence in the East.

In reference to the question of its contagiousness, he was inclined from all that he had seen to answer it in the negative. He regards the disease as *endemic*, not only in Egypt, but also in Syria and along the whole coast of Turkey. He attributes its diffusion to the filth of the towns, and the uncleanliness of their inhabitants. The better classes, and all those who practise daily ablutions and live temperately, are observed often to escape its attacks entirely, although the pestilence is wide-spread and very fatal all around.

Notwithstanding, however, that such are his opinions, *M. Renoult* approves of the institution of Lazarettoes; but he thinks that they are useful chiefly as a means of quieting alarm and apprehension.

*M. Villermé* acknowledged that, as he had not himself seen any cases of genuine plague, his opinions upon any subject connected with it must be, in a certain manner, merely speculative. He could not however assent to the doctrine that the plague was attributable to the filth and uncleanliness of the towns and of their inhabitants.

M. Renoult had alluded to the circumstance of the streets being unpaved and thereby becoming wet and muddy, when the heavy rains fall or the rivers overflow their banks; and he proceeded to shew how the mud, being acted upon by the high heat of the climate, passes into a state of fermentation and exhales the noxious miasma, to which he ascribes the origin and spreading of the disease.

All this will not however, explain why the plague is *endemic* in Egypt and other eastern countries, while it is unknown in other countries—for example in America—where the towns, as Vera Cruz, Carthagena, &c., are quite as unhealthy and as badly appointed.

*M. Recamier* suggested that, in all discussions and disquisitions on the contagiousness not only of the plague but also of other better known fevers, the question is usually considered by medical men in too absolute a point of view. There are, he said, various ways or manners in which certain diseases are transmitted from place to place and communicated from one individual to another. For example, small-pox may be communicated by direct contact, and also by the effluvia of the disease being conveyed through the air to a distance.

If we study the history of typhus fever, we shall find that its miasm may spread from a single isolated point, and propagate itself *de proche en proche*, until it becomes very widely diffused. How strikingly is this fact proved by what, we are told, took place many years ago at Oxford, during the assizes held there. Certain criminals, who happened to be labouring under typhus fever at the time, were brought into court for trial; the miasms or morbific effluvia from their bodies must have been diffused for a considerable extent around, for some of the judges sickened with the disease on the very evening of the trial.

If we read the history of the plague of 1720, written by Bertrand and Chicoineau, we see another example of a disease proceeding at first from an isolated point, and gradually propagating itself *de proche en proche* all around; and if we pass from thence to Moscow, we shall find that the pestilence there followed the same course. In short, whatever may be the nature of the origin of the plague, typhus, yellow fever, and such diseases, it appears that, when the morbific miasms are once and fairly developed, they create an atmosphere of infection, which may contaminate those who respire it. M. Recamier concluded by again pointing out how necessary it is to distinguish the origin of these diseases, and their properties or powers, after they are once fairly developed.

*M. Rochoux* followed M. Recamier, and expressed his entire concurrence in the doctrines laid down by his experienced friend. He was of opinion that there cannot be a reasonable doubt that typhus is *eminemment contagieux,* and is directly communicable from one person to another.

That there is a difference between the contagious qualities of different diseases is abundantly obvious. Thus an atom of poison is sufficient to reproduce the variolous and the syphilitic diseases. On the contrary, a certain amount or dose of the morbific miasm is necessary to reproduce typhus fever; and in this respect the plague may be associated with it. Savaresi has most distinctly stated that the plague is transmissible from one individual to another; and Clot Bey has acknowledged his error in asserting that the disease was not contagious, and has retracted the assertion, which he formerly made. M. Rochoux however admitted that few febrile diseases were so actively contagious, as has been generally supposed; and the extravagance of the fear of the many has had the effect of driving a few observers into the opposite extreme—that of denying their contagiousness altogether. It would appear, however, that he (M. R.) is opposed to the establishment of Lazarettoes; as he concluded by saying, *"all that is necessary to extinguish the disease is to scatter the sick about;"* (il suffit pour eteindre la maladie de disseminer les malades).

*M. Chervin* quite assented to the opinion of MM. Recamier and Rochoux, that typhus fever is contagious only in certain conditions; or, to express his sentiments in other words, that the disease is not communicable directly from one person to another, but is only transmissible in the way of infection when the atmosphere around becomes loaded with the miasms which exhale from the bodies of the sick. Like the latter, he too was opposed to the sanitary measures usually employed to arrest the progress of the disease. These measures, he said, are pernicious, and withal useless; as we have only to dilute and disperse the morbific miasm, by separating and scattering the patients, to get rid of the disease altogether. To regard a disease as non-contagious, and yet to argue in favour of Lazarettoes is evidently a self-contradiction;—a dilemma into which M. Renoult has wilfully allowed himself to fall. He approves of such establishments on the ground of quieting popular alarms; and I, on the other hand, disapprove of them as tending only to increase and keep up such apprehensions.

*M. Bouillaud* expressed his surprise at hearing any member of the Academy talk of the contagion of typhus fever. It is indeed admitted by all, that whenever a large number of persons, especially if labouring under febrile disorder, is crowded together in an unhealthy space, the atmosphere becomes *fort peu agréable;* but there is a mighty difference between such a state of things and genuine contagion.

From all that I have seen, I verily believe that there is not a single indubitable instance of, what has been called, typhus being communicable from one person to another. When such an occurrence seems to take place, it is always referrible to the atmosphere of the place having become vitiated from unfavourable circumstances. Such being M. Bouillaud's opinion, we are at once prepared to expect that he will unhesitatingly condemn the establishment of lazarettoes.

*M. Larrey* having expressed his decided opinion, in opposition to that of M. Renoult who had been under his orders, that the plague is infectious, and having alluded to the cases of several medical men who had lost their lives from acting upon the opposite sentiment, M. Renoult remarked in reply that, in all the instances to which M. Larrey had alluded, the constitution of the patients had been enfeebled and exhausted either by excessive fatigue or by intemperance and other irregularities; and he therefore considered that the argument was not so unanswerable as his senior imagined it to be.

*M. Castel* was of opinion that much of the disagreement among medical men on the subject of contagion arose from discussing the question too absolutely and exactly. From what he had himself seen of the yellow fever of the new world, he was quite convinced that no one could fairly say either that the disease was always contagious, or that it was never so.

The quality of contagiousness is not essential to perhaps any fever; it is merely an *epiphenomenon*, or adjunct character, which may be present or not, the disease remaining the same in either case.

---

### Discussion at the Royal Academy of Medicine on The Ætiology of Club-feet.

*M. Cruveilhier*, in his report on a Memoir of *M. Martin*, expressed his assent to the theory which the author had proposed to explain the occurrence of this deformity, and which is based upon the idea that it is attributable to irregular and unequal pressure of the uterus upon the feet of the fœtus, in consequence of a deficiency of the amniotic fluid. He (*M. Martin*) had very ingeniously adduced a considerable number of cases, which, he thought, went to prove the justness of this explanation. In all of them, according to his statement, the women experienced, about the fifth or sixth month of pregnancy, a fixed and often a very excruciating pain in the epigastric, or in one of the hypochondriac regions, according as the child lay vertically or diagonally—this pain he ascribes to the pressure of the uterine parietes against the feet of the child.

The varieties of the deformity are depending, according to this theory, on the relative situation of the one foot to the other at the time when the uterus is exerting its powerful pressure upon them. If but one limb is exposed to this pressure, there will be the deformity in one foot only; but if both suffer from it, the deformity will be double. Then too, according as the feet are in a state of flexion or of extension, the deformity will be either a *varus* or a *valgus*.

M. Martin has admitted another element into his ætiological doctrine of club-feet. He supposes that there is a certain degree of atrophy or of tardy development in the feet themselves; in consequence of this, their joints do not acquire the ordinary cohesiveness and strength, and are thus more liable to be affected by the pressure of the uterus upon them. M. Cruveilhier intimated his dissent from this part of M. Martin's theory.

The concluding part of M. Martin's memoir is occupied with a statistical table of the cases of club-foot, which he has had an opportunity of examining. In 61 cases 26 were double, and 35 were single: of these 35, 18 were of the right, and 17 of the left foot. Of the entire number, 45 occurred in boys, and 16 in girls.

*M. Capuron* related the cases of two women whom he had attended in labour, where there was certainly no deficiency of the amniotic fluid, and yet who gave birth to children affected with club-foot. He was therefore obliged to withhold his assent to the theory proposed by M. Martin to account for this deformity.

*M. Bourdon* suggested another objection, viz. that club-foot is rarely a congenital affection, but usually occurs a year or so after birth.

*M. Breschet* expressed his surprise that an able physiologist like M. Cruveilhier could give his assent to such a mechanical theory as that proposed by M. Martin. Under what circumstances can the uterus exert the alleged compression upon the fœtus? If the amniotic fluid be present, such pressure is impossible; and again, if this be discharged or is very scanty, the expulsion of the child is almost sure to follow very quickly. And even if the child is retained for some time afterwards, every physician knows that its death is inevitable.*

* These obstetrical assertions of M. Breschet are not to be implicitly relied upon.

He (M. B.) attributed this and almost every other congenital irregularity to an arrested or interrupted development of the parts affected.

*M. Velpeau*,—after renouncing all belief in the doctrine of arrested development as an explanation of congenital deformities, and denying the very basis on which this doctrine is founded, viz. that of the coalescence of the two lateral halves of the body in the median line—expressed his opinion that the idea of M. Martin regarding the ætiology of club-feet might be partially, and, to a certain extent, correct. He alluded to two instances of this deformity in cases where the amniotic fluid had been discharged a month or so previous to delivery. Although not disposed to attach much importance to the mechanical action of the womb on the limbs of the child, he was still of opinion that it may be *pour quelque chose*, in certain cases of congenital irregularity.

The theory proposed by M. Guerin, and which had met with the approval of the Academy of Sciences, seemed to him the true one. This theory, based upon a great number of observations, referred the greater number of monstrosities, as well as of congenital deformities of the joints, to a foregoing organic affection of the nervous centres, and to the convulsive muscular contractions thereby induced. Add to this, that the muscles so affected are struck with a sort of consecutive arrested development, which retards their complete formation; and then you have the real and effective cause of such deformities as club-feet, &c. It is therefore to a disease in the origin of the nerves, or in the nerves themselves, that most instances of monstrosity and of congenital deformity are attributable.

Sometimes we find that the greater part of the encephalon and of the spinal marrow have been destroyed, and then all the muscles of the body are more or less rigidly contracted; at other times, a part only of these nervous centres is diseased—say the lower part of the spinal marrow, as in spina bifida, &c.—and then the muscular retraction is limited to the lower limbs. Every case of club-foot is, in my opinion, attributable primarily to some disease of the spinal cord, or of the nerves of the affected extremity. Certainly no rational physiologist can well believe that any interruption or *arrest*, to make use of the fashionable phrase, of the normal development of the member, can have anything to do with the production of club-foot. Besides that at no period of intra-uterine life is there any semblance of the deformity in the fœtus, the numerous varieties of this affection must surely quite remove it from the patronage of this theory.

We believe that the same objections are equally potent against the doctrine of arrested development, as explicable of anencephalism and other congenital irregularities.

---

### PHYSIOLOGICAL AND THERAPEUTIC EXAMINATION OF VERATRIA, BY DR. FORCKE OF HANOVER.

The seeds of the *veratrum subadilla* had been long in use as a vermifuge remedy, more especially against the tape-worm, before it was known that they acted in any other manner than as a powerful drastic purgative. They were employed also, as an outward application, to get rid of vermin about the scalp; and in some cases, when thus used in too large quantities, very unpleasant effects seem to have been produced. Schmucker and Herz had recommended small doses in various nervous complaints, as chorea, epilepsy, chlorosis, mania, &c. In consequence however of the uncertain effects of the drug, it had fallen very much into disuse, at the time when the experiments of MM. Pelletier and Caventon in France, and of M. Meisner in Germany, proved that it contained a salifiable alkaloid base of great power and activity: the name of veratrine was given to it. To M. Magendie we owe the first series of experiments to ascertain its physiological and therapeutic effects: these were recorded by M. Andral (the son) in the Journal de Physiologie Experimentale, No. 1, 1821.

A minute quantity of the acetate introduced into the nostrils excited the most violent sneezing; when applied to the throat, profuse salivation, accompanied with a sense of most irritating tickling, was the consequence. Administered internally, it produced violent colic, vomiting and purging; a large dose brought on tetanus and death. M. Magendie contented himself with recommending the external use of veratrine in chronic rheumatism, gout, neuralgia, and anasarca; but it does not appear that he had made many trials on the human subject with it.

Dr. Bardsley of Manchester first, and subsequently Dr. Turnbull of London, are entitled to the merit of having distinctly proved the powerful efficacy of this and some other cognate alkaloids in medical practice. As their works are however well known to the public, we shall now proceed to give a short summary of the researches of Dr. Forcke.

Taken internally, in the dose of a sixth part of a grain and repeated twice or thrice at the interval of a few hours, it causes, he says, a sensation of formication or tingling, and of heat in the epigastrium first, and subsequently in other parts of the body, as the limbs, feet and hands, the forehead, nose, &c. This most unpleasant feeling, as if a myriad of pins' points were kept being gently applied to the parts affected, is accompanied with a sense sometimes of heat, at other times, of cold; and the skin seems to be so very extraordinarily susceptible of the impression of either, that the slightest wave of warm or of cool air is quite painfully disagreeable. Dr. Forcke asserts, contrary to the statements of others, that the effects of the medicine administered for some time do not seem to increase beyond what are manifested during the first few days.

Along with the phenomena now noticed, there is another which is of occasional occurrence and is a very remarkable one: a pain, which has long occupied a certain part, suddenly vanishes altogether, or is replaced by one in some other part of the body. This influence seems to manifest itself *de preference* in organs which have been effected with paralysis or neuralgia.

There are some persons in whom the veratrine seems to be entirely impotent; but the number of these is few, and generally they are old people. The internal use of this alkaloid in most cases causes a certain degree of constipation of the bowels; and hence it may be necessary to have recourse to aperients during its administration.

In illustration of the therapeutic effects of veratrine, Dr. Forcke adduces the following cases.

*Neuralgia.*—Five cases are reported: in all of them, the suffering was seated in some of the branches of the trigeminus nerve. In the first three, the patients were old women, who had for many years been subject to violent and frequently repeated paroxysms of pain in the infra-orbital nerves. The external use of the veratrine—a scruple of the alkaloid to an ounce of lard—succeeded in entirely removing the disease, which had baffled all other means of medication.

The fourth case occurred in a middle-aged woman, who had for thirteen years suffered from neuralgia of the supra-orbital nerve: the attacks usually came on before each appearance of the catamenia. Frictions with the veratrine ointment were followed by *un succes durable.*

In the fifth case, which was that of a man 38 years of age, the disease affected all the branches of the trigemini nerves, and was so severe that the patient, during the paroxysms of pain, was almost bereft of his reason. The external use of the veratrine was combined with the administration of colchicum in large doses. (The issue of this case is not specified).

*Palsy.*—Nine cases of partial palsy, affecting different parts of the body, are related by Dr. Forcke in proof of the efficacy of the veratrine ointment. The first was that of a youth, who had quite lost all use of his left arm, without any appreciable cause: the limb was utterly powerless, and had become much emaciated. The internal and external use of strychnine, as well as various other methods of treatment, had been tried for a length of time, but without success. Frictions with the veratrine ointment were now employed, and ultimately the lad quite recovered the use of the arm.

We shall briefly notice another of the cases. A man of robust constitution presented the following appearances, when he first consulted M. Forcke. The left side of the face was much swollen; its muscles were in a state of complete relaxation; the eyelids were motionless, and the left one was always depressed, so that the eye was constantly exposed. The consequence of this exposure was that the conjunctiva had become much thickened and inflamed, and the sight of the eye was nearly abolished. The branches of the inferior maxillary nerve were affected with periodical paroxysms of most acute pain. The right side of the face was much contracted and wrinkled, the angle of the lip and the wing of the nostril being drawn upwards. The hearing too was dull, and the patient experienced a considerable degree of impediment in his speech.

The internal as well as the external use of the veratrine was commenced; but the amendment proceeding very slowly, Dr. Forcke applied three grains of the alkaloid to the denuded dermis over the places, where the facial nerve and the first and second branches of the trigeminus make their escape from the cranium. Severe pain and irritation were induced: three days afterwards, another grain was applied. The patient began to move his eyelids, and the paralytic state of the face became gradually less and less. The patient, contented with the improvement he had already made, ceased his visits to Dr. Forcke.—*L'Experience.*

The preceding notice of Dr. Forcke's "*Physiologisch-therapeutische Untersuchungen ueber das Veratria,*" quite confirms all that we have previously said, in this Journal, respecting the medicinal powers of this alkaloid. That it is a most active and potent remedy, admirably calculated to relieve many cases of neuralgic suffering, is a *fact* that cannot be disputed by any one who has given a fair trial to it, provided the remedy be genuine and it induces its remarkably peculiar effects on the body. It is however proper to observe that, in some cases, where it has succeeded in relieving the most intense paroxysms of facial neuralgia, this happy result has proved to be of only temporary duration. Still it is of no little importance to be able to mitigate, if not to cure, pain.—(*Rev.*)

---

INCONTINENCE OF URINE SUCCESSFULLY TREATED WITH THE NUX VOMICA.

The attention of medical practitioners has of late years been called to the very decided effects of this potent remedy in stimulating the palsied fibres of the urinary bladder.

Signor Cerchiari has published two very satisfactory cases treated in this manner. In one of these, the incontinence was attributable to the contusion of the neck of the bladder by the passage of the child during parturition. The patient could not retain her water night or day. The following pills were prescribed.

Take of extract of Nux Vomica 8 grains, Martial æthiops and simple syrup, as much as may suffice to make a mass, to be divided into 24 pills. One of these to be taken three times in the course of the day. A perfect cure was obtained in a fortnight.

The second case occurred in a youth, 19 years of age, who had been subject from his infancy to involuntary discharge of the urine during sleep. The above pills were prescribed; and a complete cure was obtained in less than fourteen days.—*Bulletino delle Scienze Mediche di Bologna.*

---

### On the Use of Chlorine Inhalation in Catarrh.

Dr. Toulmouche, a physician resident at Rennes, and one of the medical officers of the *Maison de Detention* there, has been engaged for the last five years in ascertaining the virtues of chlorine inhalation in the various forms of bronchitic disease.

He usually commences with advising ten or twenty drops of the concentrated solution to about half a pint or so of warm water, and he increases this quantity, by daily adding five drops or more, to sixty or even eighty, according to the susceptibility of the invalid. The solution, it is to be remembered, should always be kept secluded from the light.

The chlorine inhalation he recommends even in acute catarrh, provided there be no co-existent pulmonary engorgement or tuberculous induration. As long as the disease is limited to the mucous surface of the air-tubes, the practice is not only safe, but salutary; and it never seemed to aggravate the inflammatory action.

When however the cough and dyspnœa were very great, or when the idiosyncracy of the patient was opposed to the use of the chlorine, Dr. Toulmouche strongly recommends the inhalation of the steam of hot water in which opium or belladonna has been infused. Blisters applied to the thighs are also highly useful in such circumstances.

As a useful expectorant drink, Dr. T. praises a solution of the chloride of soda; ʒss—ʒj. in a pint of water. The unpleasant taste and smell of this solution are however objectionable to most patients.

Before we give a decided opinion on any case of old catarrh or chronic bronchitis, and before therefore we decide upon any line of treatment, it is most important that we satisfy ourselves as to the real nature of the existing disease, and as to the complications which may happen to be present at the same time. We have already alluded to the not uncommon complication of pulmonary engorgement or tuberculization—two morbid states of frequent occurrence, and on which a bronchitis is very often engrafted. As a matter of course, whenever such a state of things exists, our prognosis must be much more guarded, not only as respects the issue of the case, but also as far as the mere temporary alleviation of present distress is concerned.

Another frequent complication is asthma, depending upon emphysema of the lungs. Whenever this state of the pulmonary tissue exists, we can never hope for an entire restoration of the patient's condition.

A dilated state of the bronchi also is of not unfrequent occurrence in cases of chronic catarrh: we may likewise add partial or circumscribed pneumonia in a latent form, pleuritis, effusion into the chest, &c. &c.

Dr. Toulmouche informs us that chronic catarrh *regne habituellement* in the Penitentiary or central house of detention at Rennes; and that it and pulmonary consumption are by far the most frequent diseases in its infirmary. He attributes this to the dampness of the locality, to the cold humid state of the workshops in the Penitentiary, and to certain unwise regulations on the clothing of the inmates.

The number of cases, in which Dr. T. has used the chlorine inhalation in the course of four years and a half, has been 309; of these 228 occurred in females and only 81 in males. This appears certainly rather strange, as from most clinical tables, the number of men affected with the various forms of catarrh is almost always much greater than that of women. M. Andral distinctly states that the disease is most common in the male sex.

Of the 228 cases, there were 141 of acute, and 63 of chronic bronchitis; in four, the disease was complicated with pulmonary emphysema, and in the remaining 22 with phthisis.

More than one-half of the cases were cured in from two to nine days, after commencing the use of the inhalation. As might be expected, the cure of the chronic disease was more tardy than that of the acute form: on an average it occupied from sixteen to thirty days or so.

Dr. T. has given the reports of numerous cases, to shew the efficacy of the chlorine inhalation in the various forms of bronchitis.

It seems, however, unnecessary to extract any of them. Suffice it to say, that according to these statements, the remedy seems to be a very potent and useful one in checking the profuse expectoration, and in restoring a healthy action of the bronchi.

In conclusion, it may be stated that Dr. T. has no faith at all in any sort of medicated inhalations in genuine pulmonary phthisis; and in the truth of this opinion we (Rev.) believe that almost all practical men will agree.—*Gazette Medicale de Paris.*

---

### REMARKS ON, WITH CASE OF, PNEUMO-THORAX.

The following case lately occurred in the practice of M. Chomel, at the Hôtel Dieu.

A man, affected with pulmonary tubercles in a state of softening, was suddenly seized with a violent pleuritic pain or stitch, which caused extreme difficulty in breathing, and considerable febrile action in the whole system.

On examining the chest, it was observed to be altogether fuller and more capacious than it had been, and at the same time to be much more resonant on percussion, while the respiratory murmur had become much more indistinct.

When lecturing upon this case, M. Chomel took occasion to state that, in his opinion, the disease of *Pneumo-thorax* is never idiopathic,—or, in other words, that air is never secreted from the pleural surfaces—but is always the result of a communication between the air cells of the lungs and the bag of the pleura. Such a communication may take place in one of two ways; either by the ulceration of a vomica outwards, as in the present case, or in consequence of a purulent effusion into the pleural cavity being followed by an ulceration at some point of the lungs.

He stated at the same time that he took the same view of tympanites abdominalis—the effusion of air being, according to him, always the result of an intestinal perforation.

Such a perforation may have taken place either from within outwards, as is occasionally the case in some cases of typhus fever, or from without inwards, as now and then happens in consequence of a purulent collection in the abdominal cavity. An instance of this sort occurred very recently in M. Chomel's clinique in a woman, who died from an immense abscess in the pelvis: ulceration had taken place at one point of the large intestine, and had penetrated through all its coats, except the mucous one.

It is necessary to distinguish the peritoneal tympanites, which we not unfrequently meet with in dissection, when there is certainly no perforation of the intestines, from that *alleged* or *presumed form* of the disease, which has been attributed to the secretion of air during life: it is entirely owing to incipient cadaveric decomposition. M. Chomel mentioned a very remarkable instance of this cadaveric tympanites, which he recently met with. A restaurateur in Paris, who was immensely fat, but seemed to be in very good health, was most unexpectedly found dead in his bed. The body was examined 30 hours after death. The season was Summer. No sooner were the abdominal parietes divided, than a loud explosion, which M. Chomel compares to that produced by the discharge of an air-gun, was heard; so violent was the rush of the confined air from the aperture which had been made into the cavity of the abdomen.

But now to return to our case of pneumo-thorax.

On the following day, after the presumed rupture of the vomica, and the communication between the air-cells of the lungs and the pleural cavity had taken place, the expansion and also the resonance of the thoracic parietes were found to have considerably increased.

In proportion as the pneumo-thorax was more decided, the auscultatory signs became more and more distinct and decisive. At the lower part of the chest a sound, analogous to the amphoric bruit, was perceptible; and more externally a distinct metallic tinkling was to be heard. Over the scapular region a *bruit de secousse*, such as is caused by striking a drum with the finger, was audible when the patient spoke.

Along with these symptoms, there was extreme anxiety and difficulty of breathing, amounting to orthopnœa, &c. and, as we have mentioned above, the chest was remarkably resonant on percussion.

The treatment which M. Chomel adopts in almost all cases of internal perforation, whether of the thoracic or of the abdominal viscera, consists in the exhibition of opiates, until they produce a decided narcotism of the system. The object is not only to quiet the pain which is almost always present, but also bring on a state of *inertia* of the whole system, so as to permit Nature to exercise her own medicative and reparatory efforts.

From the results of several cases of presumed intestinal perforation, this treatment certainly seems to be by far the most advisable.—*La Lançette Française.*

---

### Observations on the Perforation of the Intestines by Worms. By Dr. Mondiere of Loudun.

We may premise the following remarks by stating that a perforation of the intestines by worms may take place in two ways—*either* by the worms lodged within the alimentary canal making a way outwardly into the cavity of the abdomen, or fairly through the abdominal parietes, *or*, by the bursting of an '*abscès vermineux*,' (developed, as such usually are, in the inguinal and umbilical regions) into some part of the canal.

[This distinction is not very perspicuous, the language of the writer being obscure.—*Rev.*] As the main object of this memoir is to afford proofs of these occurrences, we shall at once proceed to mention a few illustrative cases.

A young girl had been for a length of time troubled with worms, many of which she had passed at different times, not only from the rectum, but also from the mouth. After a violent attack of colic, induced by the exhibition of some vermifuge medicines, a small swelling made its appearance in the left iliac region. At first it was attended with a heavy dull pain; this was afterwards replaced by a most annoying sense of pricking.

The swelling became gradually larger and softer, as well as more prominent; it was opened, and a quantity of pus escaped. But what was our surprise, when we observed a *lumbricus* worm, five inches and a half long, discharged at the same time! Notwithstanding this, the abscess healed up, and the patient experienced no subsequent inconvenience. It is particularly to be observed, in reference to this case, that in the discharge from the abscess there was not the slightest trace of any intestinal matter in it.

Dr. Mondiere is of opinion that, in such cases, we may fairly suppose that the worm has not made any regular ulcerated perforation in the parietes of the intestine, but has merely separated (*ecarté*) the fibres of the canal, and that thus no fistula has been occasioned.

Medical men have been much puzzled to explain such an occurrence as the preceding. Thus M. Destrez, who has recorded a case very similar to ours, asks: "Whence are we to suppose that the worm proceeded? It must indeed have been developed in the intestines; but how did it escape, without causing a perforation of their parietes, and, if so, without being attended with the symptoms of intestinal rupture? It may possibly have come from the appendix vermiformis, whence it might escape without such serious consequences."

That M. Destrez's conjecture respecting the appendix vermiformis cannot be received is manifest from the tumors, of which we are now treating, being observed frequently on the *left*, and not on the *right*, side. In the following case, the swelling was in the umbilical region.

A girl had long suffered from attacks of *verminous colic*; an abscess formed near the umbilicus; along with a copious discharge of purulent matter, a round worm, dead, and between four and five inches in length, escaped from the orifice. The abscess however gradually healed without any unpleasant symptoms.

Dr. Mondiere alludes to a case which occurred many years ago to M. Chailly (and which is recorded in the *Nuovo Giornale della piu recente Litteratura Medico-Chirurgica d'Europa*, for 1795), of a young child, in whom an abscess formed in the hypogastric region giving exit to a *strongylus*, and who recovered perfectly.

In the *Journal des Progres*, for 1834, is related the case of a negro, 11 years of age, in whom after a most severe attack of *verminous dysentery*, during which a great many *lumbrici* were evacuated, there formed near the umbilicus an abscess, which gave exit to a dead *lumbricus*.

Dr. Mondiere here alludes to the occasional, but certainly rare, occurrence of *entozoa* making their way from the intestines into the urinary bladder, through the parietes of both viscera; and he explains the process or manner in which this is done, by referring to his previous observations respecting the gradual *ecartement* or separation of the fibres of their different coats effected by the animalcula, without this being necessarily accompanied with an ulcerated perforation.

We do not intend, however, that it should be supposed that an ulcerated opening through the intestines is never occasioned by the escape of worms from their cavity. That such is really the case sometimes is made manifest by numerous instances on record. Take for example the following case.

A girl, 12 years of age, died dropsical and in the last stage of scrofulous disease. On dissection, the small intestines were found to be perforated in five or six different places; and hanging from these apertures were observed *lumbrici* more or less completely protruded. Other worms, of the same description, were found in the abdominal cavity, floating in the serous effusion which was there. The apertures in the intestines corresponded exactly with the size of the lumbrici.*

*Rem:*—Dr. Mondiere seems to think that, in this and in similar cases, the perforation took place after death: if so, we cannot regard it as an example of *ulcerated* opening.

Dr. M. sums up his observations in the following propositions:—

1. Intestinal worms may make to themselves a way through the walls of the bowels and even through the abdominal parietes, by merely separating, and, as it were, splitting the fibres of the intervening tissues, and not, as has been commonly imagined, by gnawing and corroding the parts.

---

* Journal der Pract. Heilkunde, 1834.

2. The immediate obliteration of the passage, through which the worm has escaped, is to be attributed to the contractility of the intestinal fibres, and to the closure thus effected.

3. Cases of the escape of intestinal worms have been usually noticed in persons, who have been annoyed with vermination, and who may have voided many of those entozoa both upwards and downwards.

4. We are ignorant of the cause which leads these animalcules to move about: in some cases it would seem to be owing to the irritation excited by acrid anthelmintic medicines.

5. The symptoms, which have been noticed to be induced by the escape of worms through the intestinal parietes, are usually a painful pricking or puncturing pain at one fixed part, and then the formation of a phlegmonous swelling over this part; the swelling becomes gradually larger and softer, till at length it breaks or is opened, and the worms are discharged along with the pus.

6. These swellings may form at any part of the abdominal circumference; but most frequently they are observed either in the umbilical or in one of the iliac regions.

7. Cases of this sort generally do quite well; the abscess healing up without any unpleasant symptom, or sign of intestinal lesion. In some cases, the worms, have made their way into the urinary bladder, without causing any extravasation.

Of a much more serious character is the *second* description of cases, in which intestinal worms make an opening for themselves through the parietes of the bowels. In this, the worms having been congregated and agglomerated together at one point, at length excite inflammatory action in the part; the intestine becomes adherent to the opposed surface of the abdominal parietes; an abscess is formed, and when this bursts, the purulent matter which escapes is blended with worms, and also with the other contents of the bowels. The last named circumstance—the admixture of fæculent matter with the discharge—establishes a strong line of demarcation between this and the preceding description of intestinal perforation. The former, we found, was attended with comparatively very little seriousness or danger; the perforation seldom or never giving rise to any alarming symptoms. The latter is necessarily of much greater moment. Extravasation of the intestinal contents into the cavity of the abdomen is indeed of rare occurrence; this calamity being prevented by the adhesion of the intestine to the abdominal parietes; but a dangerous fistula, communicating with the gut, may be established, and may remain open for a great length of time. In many cases however, which may seem alarming at first, the abscess, which has given exit not only to worms but also to fæcal matters, will be found to heal as rapidly as if it had been a simple phlegmon.

*Case.* A woman, 33 years of age, who had been annoyed with intestinal worms more or less from her infancy, found, after suffering for some time from colicy pains in the right flank, a tumor in her right groin; the swelling increased gradually in size and prominency, and, as it became more soft and pointed, she felt a strange moving or creeping sensation in it. No fluctuation could be detected in it, when Dr. Mondiere first visited this patient. In the course of a few days, however, the apex of the tumor having become gangrenous, a bistoury was plunged into it. A quantity of ordinary pus was discharged. On the following day a dozen of worms (ascaris lumbricoides) issued from the wound; some of these were still alive and moved about freely. During the next three or four days, seventeen more worms were discharged; and along with them some fæculent matter. Notwithstanding this unpromising state of affairs, the abscess healed slowly but kindly, and the patient's health, which had never suffered much, was completely restored.

*Case* 2. A child, seven years of age, had been subject, for upwards of a twelvemonth, to attacks of sharp abdominal pain about the umbilicus. A phlegmonous swelling made its appearance at length in this region; it gradually became softer and more prominent, and burst. In the purulent matter, which flowed out, was found a dead *lumbricus* worm. A fistula remained open for a great length of time; and at length upwards of forty living worms were passed *presque tout à coup*. The fistula continued open, and gave exit to eleven more worms. It had not healed at the date of the report, but had acquired all the characters of stercoral fistula; the attacks of colicy pain had however ceased.*

*Case* 3. A boy, 14 years of age, after suffering for upwards of a twelvemonth from attacks of violent colic, general decay of health, &c., had a swelling form near to the umbilicus. It was attended with a smart burning and pricking pain; and gradually it became more and more prominent, and at length burst, giving exit to some healthy purulent discharge. On the fifth day after this occurrence, the head of a *lumbricus* woım was observed projecting from the wound; it was pulled out without difficulty. During the following eight days, other three worms were extracted: after the escape of the last one, a yellowish-coloured and fetid matter, which was regarded as fæcal, flowed out. After the lapse of a considerable space of time, another worm, alive and larger than any of the preceding ones, escaped from the wound. This was the last; and the tumor and discharge now began to diminish very sensibly: at length, the wound healed entirely, and the patient's health was completely re-established.†

M. Mondiere has related several other cases, all illustrative of the formation of these *verminous* or *worm* abscesses. He sums up his observations in the following summary.

1. The formation at some point of the abdomen—usually the umbilical or inguinal region—of a tumor which on bursting gives exit to worms, is of occasional, and not very rare, occurrence.

2. These tumors are observed to occur most frequently in those who are and have been much troubled with vermination: hence most cases occur in young persons.

3. It is probable that, in the cases of which we are at present speaking, the worms have congregated at one part of the intestinal canal; that this part becomes distended and then inflamed; that the intestine forms an adhesion to the opposed surface of the abdominal peritoneum; and that ultimately the process of suppuration and of ulceration outwardly is established.

4. The formation of these tumors is always preceded and accompanied by the existence of sharp lancinating or vibratory pains in the part where the tumor forms: this symptom is in some degree characteristic of the nature of the tumor.

5. In many cases, the health of the patient has suffered for a great length of time from obscure intestinal disease, before the tumor has become at all developed.

6. After the bursting of the abscess, a genuine intestinal fistula remains: this usually continues open until all the worms are discharged; and then it heals up without further trouble.

7. The *ascaris lumbricoides* is the worm which in almost every case is discharged. We know however of one or two instances where the worm was a *tænia*.

8. In most cases, there are evident signs that the fistula communicates with the cavity of the intestinal canal. Occasionally, however, it would seem that

---

* Il Filiatre-Sebezio 1837, and Gazette Medicale 1837.

† Archives de Medecine, 1838.

the worms, on escaping from the bowels, become enveloped in a cyst, and that the aperture in the tube has healed before the outward abscess bursts.

9. The treatment of such cases is extremely simple: we should do very little, and not interfere with Nature in her efforts to discharge the offending matters first, and then to close up and heal the fistula which has been formed.—*L'Experience.*

---

### Expulsion of a long Loop of the Intestinal Canal by the Rectum.

Three years ago, Signor Vulpes presented to the Medico-Chirurgical Academy of Naples a portion of small intestine, three feet in length, which had been passed from the rectum. The patient, a woman, who had been affected with volvulus from intus-susception, recovered perfectly—except that she was rather subject to flatulent distention of the bowels after food, and that there remained a hardness between the right hypochondriac and the epigastric regions—and she continued to enjoy very good health until last year, when she was seized with enteritis and died.

The dissection of the body was performed with much care, and the pathological specimen obtained was presented to the Academy.

The abdomen contained two pints of a sero-purulent effusion. At the distance of 27 inches from the pylorus, it was found that there was an adhesion of the two portions of intestine which had been severed or divided during the attack of volvulus. These two portions were united together by their outer or peritoneal face, and the continuity of the canal had thus been perfectly re-established. The jejunum was much expanded or dilated, and its parietes were considerably thickened and more muscular than usual, above the seat of union, so that it almost resembled a second stomach—and on slitting the gut open, it was found that, at the point of union, the diameter of the tube was very much contracted.—*L'Observatore Medico di Napoli.*

---

### Case of remarkable Pulsation in all the Superficial Veins of the Arms and Neck.

A man, 26 years of age, of a lymphatico-scrofulous temperament, and of a rather feeble constitution, was admitted into La Charité Hospital on the 30th of July, 1835.

In 1828 he had had a severe pleurisy of the left side; and in 1830 he had a smart attack of acute rheumatism.

For ten days preceding his admission into the hospital, he had been labouring under diarrhœa, frequent vomitings, pain in the right hypochondrium, and general feverishness. There was a very appreciable tumefaction of the left hypochondrium, attributed to an enlarged state of the spleen. The respiratory functions did not appear to be much affected. The treatment consisted in the administration of a tartarised potion, the application of leeches to the anus, and the use of *bouillon coupé.*

During the first fortnight of the patient's residence in the hospital, there was no amelioration of the symptoms: the action of the bowels was irregular—at one time relaxed, and at another confined—and the febrile irritation had become more and more decided. The pulsations of the heart were now very energetic and vibratory, and were accompanied with a blowing and occasionally also with a rasping sound during the first beat: blood had been thrice drawn, and at each time it was buffy. At this period it was observed that there was a distinct pulsatory movement in all the superficial veins of both upper extremities, and in the external jugulars, more especially in that of the right side. This venous pulsation was more sensible in the dorsal veins of the hand than in those of the fore-arm; but it was strongest and most distinct in the jugular veins. Observed in relation to the arterial pulse, it was observed that this always preceded the venous one. The blood during venesection flowed out *per saltum*.

On the 16th of August the following was the state of the symptoms. The bowels were still more or less purged; the pulse was rapid, large, and strong; the first *bruit* of the heart was attended with a slight blowing sound, and a distinct purring tremor (*fremissement cataire)* was audible over the apex of the sternum and over the subclavian trunk; the venous pulsation was generally sensible to the finger.

The patient gradually sunk, and died two days afterwards.

*Dissection.* The meninges of the brain were highly vascular, and exhibited other signs of decided sub-inflammatory action. There were very extensive adhesions, and a considerable effusion of opaque serosity, between the pleuræ on both sides. Both lungs were highly congested, especially in their lower thirds, and were feebly crepitant. On being divided, a quantity of sanguinolent frothy serosity flowed out, and, here and there, the pulmonary tissue exhibited some *apoplectic points,* when the effused blood seemed to be blended with the proper substance of the lung.

The pericardium was healthy; and the heart was of the normal size, although somewhat laxer in texture than usual. Its *tricuspid* valve was rather thickened at one or two points, and exhibited a fibrinous deposition; and, near the opening of the pulmonary artery, there was observed a pseudo-membranous layer of a yellowish-white colour, as if it were formed of concreted pus. The *mitral* valve was hard, thickened, with irregular nodulated edges; the aortic valves were singularly altered, much thickened, opaque, friable, *obstruant completement la lumiere de l'aorte.*

In the abdomen, a small quantity of citron-coloured serum was found; the spleen was much enlarged, weighing more than a couple of pounds, indurated in some and softened in other points, and on the whole highly congested with dark blood. The gall-bladder contained numerous dark-coloured gall-stones, and on the surface of its parietes were observed several distended varicose veins. The liver presented a great number of abscesses of various sizes and degrees of maturation. (*Note.*—There had never been any symptoms of jaundice during the life of the patient.)

*Reflections.*—A considerable number of authentic cases of venous pulsations —in some of which they were much more general and extended than in the preceding observation—are on record. This phenomenon was noticed,

1. In a young girl who died with hydrocephalic symptoms.—(*Haller's Disputationes*).

2. In a case where vertigo and general pains in the limbs were the most marked symptoms. The phenomenon was observed for three days only, the patient rapidly recovering.—(*Journ. der Pract. Heilk. Sept.* 1815.)

3. In a man who suffered from syncope, palpitations, and dyspnœa. The dissection did not reveal any satisfactory cause of the symptom.—*(Archiv. fur. Medic. Erfahrung,* 1822).

4. In a soldier, who was affected with a *paralysie lente* on the right side, which had commenced on the 1st of December. On the 23rd of January, after a slight amendment, the pulse was full and hard, but not much quickened, the skin was hot, the face flushed, and all the superficial veins of the body and limbs were observed to pulsate—this phenomenon lasted for five days. The skin in almost every part was raised and depressed at each pulsation: even the eyes and the tongue *battaient*. On the 30th, there was coma; but the venous pulsations had ceased: they re-appeared two days afterwards, and the patient died on the following day.

On dissection, " the middle semilunar valve of the left ventricle was found to be converted into an osseous concretion, so situated that it obstructed in a great measure the bore of the aorta. The lungs were much congested."—(*Journ. Complementaire, Juin* 1835).

5. In a case of epidemic fever, the venous pulsation was observed for twenty-four hours.—(*Dublin Hosp. Reports, Vol.* 4).

6. Dr. Elliotson mentions the case of a young lady, labouring under chronic bronchitis, in whom all the veins of the hand and fore-arm were observed to pulsate synchronously. The patient recovered.

7. Dr. Ogier Ward has recorded another example of this rare phenomenon. A woman, 30 years of age, was admitted into the Wolverhampton Dispensary with symptoms of feverish malaise. The pulse was quickened, the breathing hurried and attended with cough. A week after this data, she miscarried; and, three days subsequently to this event, the dorsal veins of both hands were observed to be much distended and to pulsate very strongly. These pulsations were quite appreciable in the digital veins, in which the blood seemed to have a florid arterial hue. They extended up as far as the middle of the fore-arms, and were not stopped by pressure on the veins, unless this was made below, or *distad* from, the point where the experiment was made. These pulsations were isochronous with the arterial pulse, which at the time was hard, incompressible, and frequent. The phenomena lasted for three days; and the patient recovered perfectly.

A curious occurrence was observed in this case—castor-oil did not act as a purgative, but exsuded from almost every part of the body.

With regard to the *rationale* of venous pulsations, physiologists are uncertain whether the phenomenon is to be attributed to the action of the left or of the right ventricle of the heart—in other words, whether it is to be considered as the result of the pulsation of the arterial tubes being continued through the capillary vessels and thus reaching the blood in the veins, or whether it is caused by a retrograde pulsatory action of the right ventricle, communicated backwards from the larger to the smaller veins.—*Journal Hebdomadaire.*

---

### The 'Soupe a la Minute,' recommended by Baron Percy.

The following letter from Dr. Valissiere of Metz appeared recently in the Lançette Française.

"Having just finished reading the account, given by the Surgeon-General Baudens, of the expedition against the city of Constantine, it has occurred to me that it may prove useful to recall to the attention of the public, and more especially of military men, the receipt for preparing a 'soupe à la minute,' so much recommended by our distinguished countryman Baron Percy.

Six pounds of butter are to be put into a large saucepan; this is to be fried with a handful of onions cut small, and with the same quantity of *aulx* (what are these?). The mass is to be well stirred about with a spoon, and as much good flour is to be gradually added as the melted butter will absorb. To incorporate the two better together, we may add a pound or so of olive oil to the mixture. A sufficient quantity of salt and pepper is to be added, to give flavour to it, and to fit it for keeping. As the mess cools, the stirring is to be continued very steadily; and when it is quite cold and stiff, it should be preserved in pots or tin-cases.

The above quantity of materials will suffice to make 45 basins of soup. A piece of the size of an egg may be dissolved in three pints of water, and will furnish a very palatable and nourishing aliment.

Such is the substance or material recommended by Baron Percy for soldiers, who are about to start on a march, where they may find difficulty in procuring food. Each soldier may carry a supply with him sufficient to last for a week or two; and all that is necessary for the preparation of the soup is water and a fire to heat it with. The soup thus obtained is much preferable to that made with most of the dried gelatine, which is sold now-a-days, but which, according to the experiments made by MM. Donné and Gannal, is very far from being nourishing."

---

### REMARKS ON SOME CURIOUS CASES OF SIMULATION.

Some of the French journals have lately been astonishing their readers with an account of a woman, who 'although fresh and healthy, had not touched food for a year and eight months, nor had any excretions during all that time, and who yet was suckling an infant six months old!' This woman was first under the care of M. Caillard, and subsequently of M. Magendie, at the Hôtel Dieu in Paris, where she still remains. We, says the reporter, have repeatedly seen her, and from the very first suspected an imposture: and so it has proved. When admitted into the hospital last September, her tale was that, for the preceding year and a half, she had not tasted any food, and that she had acquired the power of living without eating. For the first few days after her admission, she certainly did not take any nourishment, leaving untouched all food, fluid as well as solid, that was left within her reach.

She was then put into a small room by herself and various articles of diet,—all of which had been previously weighed and measured—were left at her disposal. It seems that she had resisted all the cravings of appetite for eight days; but on the ninth day she began to eat, and has continued to do so ever since. On examining the mattrass, numerous pieces of dry feculent matter were found concealed in the wool. Here then was an end of this *extraordinary* tale, which some of the journalists were making such a fuss about.

If we now ask ourselves, what possibly could have been the motive of this woman's conduct, and what interest was to be served by this most strange imposture?—perhaps the only answer we can make is, that it was the love of notoriety, of being spoken about, and, in short, of being the subject of curiosity to others. This in truth is a very strong feeling in many minds, and will often impel the vain and foolish to extraordinary acts of self-punishment.

Perhaps it is this motive, more than any other, which has caused so many young females to be misled by the practices of the animal magnetiser, and to simulate so many fantastic feelings and sufferings. But to drop any further allusion to these, it may be amusing to mention briefly another case, similar in many respects to the one, we have already recorded, of the woman in the Hôtel Dieu.

About fifteen years ago, there was a woman in the wards of La Charité, who *it was said,* had not voided by the natural passages 'le moindre atome' of fæcal matter or of urine, for the space of two years. The former, it was alleged, was evacuated by vomiting, and the urine partly in this way, and partly by oozing from the umbilicus. The truth of all this was very generally believed, at the time, in the hospital, both by the pupils and by Professor Leroux. It was even published by M. Nysten, in one of the journals, as an extrordinary case of aberration of the normal functions! It was indeed quite true that the patient was seen to vomit every day, a portion of fæcal matter; and she kept a piece of sponge to wipe away the urinous moisture from the umbilicus. No person suspected a trick, until old Boyer, observing that the vomited fæces were of a rounded form and consistent, remarked that they must have come from the large, and not the small intestines. This sagacious remark excited the curiosity of the attendants, and precautions were accordingly taken to guard against further imposition, if this had really been hitherto practised. The woman was made to wear a pair of drawers, which were secured round the body and ancles, so that it could not be undone, without being discovered; and the hands also were covered with gloves, which were then stitched to the sleeves of a waistcoat. An attendant remained in the apartment all night.

She repeatedly indicated great uneasiness, complained of pain, and kept her hands applied to the stomach; still she asserted most positively that she had no desire to pass either urine or stool; but that she felt the severe pain which always preceded the oozing of the urine from the umbilicus.

In the morning her distress was extreme, and the abdomen was found to be exceedingly distended. In the course of the day, 'elle n'y put plus tenir;' for she urined so abundantly that the mattrass of the bed was wetted through and through. It was now discovered that she had concealed under the bolster a quantity of dried feculent matter; portions of which she had no doubt been in the habit of swallowing, for the purpose of afterwards vomiting it up again, in order to keep up the strange imposture, which she had most unaccountably thought of practising.—*Bulletin General de Therapeutique.*

### On the Presence of Urea in the Blood.

It has long been a problem of much interest, and of no easy solution, in physiology to determine whether the secretions of the body are eliminated and prepared by the secretory organs themselves, or whether they already exist, formed in a more or less perfect degree of composition, in the blood itself, and are merely separated, and, as it were, drawn off from it at the different emunctories.

Most of the chemical physiologists have been inclined to adopt the latter of these two opinions, and it has been supposed that considerable probability has been added to it by the experiments of MM. Prevost and Dumas, wherein they discovered the presence of urea in the circulating blood of animals, from which the kidneys had been extirpated.

It would seem, however, from the recent very careful researches of MM. Mitscherlich, Gmelin, and Tiedemann, that they could not discover the smallest traces of urea in the blood of a healthy animal; although by previous experiments they had assured themselves that they were able, by chemical analysis, to discover so minute a quantity as a 250th part (of a grain?) of this animal product in blood, (on pouvait retrouver encore 1,250 d'urée dans le sang.)

M. Marchand states that he has repeated their experiments with the most minute accuracy, and has obtained the same results.

Deeming it, however, not improbable that a minute quantity of any substance like urea might be so entangled and, as it were, involved in some of the constituents of the blood, that its presence might be more or less completely disguised from the researches of the analyst, M. Marchand has performed numerous experiments to determine the question.

For example, he took 200 parts of the serum of blood, and mixed one part of urea with it; the fluid was then heated in a sand-bath, until the albumen freely coagulated, and when it cooled, the urea was sought for by the most delicate tests. But not more than one-fifth of it could be detected: how the rest of it was withdrawn or disguised, M. Marchand was not able to satisfy himself.

He also found that the fibrine and the colouring matter of the clot had a similar effect, as the albumen of the serum, in this respect: the entire quantity of the urea, which had been blended with them, could never be recovered.

M. M. invites the attention of animal chemists to this fact, as the knowledge of it cannot fail to affect very materially the results of many of their analyses.

Thus he alludes to some researches of his own regarding the presence of urea in the fluid of dropsical effusions. Whenever the quantity of albumen present in the fluid was inconsiderable, the urea was often easily detected; but when it was greater, all traces of the salt were absent.

Although, therefore, no urea can be discovered in healthy blood, it is far from being improbable that it does actually exist, in small quantity, in it.

We shall now briefly allude to the circumstances under which urea has been found in the blood; and the first, that we shall make mention of, is when the kidneys have been extirpated in the lower animals.

The names of the experimenters—MM. Prevost, Dumas, Mitscherlich, Tiedemann, Gmelin, &c.—afford a sufficient guarantee for the correctness of the statement.

M. Marchand deemed it therefore quite unnecessary to repeat the experiment; but he varied the manner of performing it in the following manner, He passed a ligature round both kidneys in a healthy sheep, (dans un mouton sain, en une seule fois je fis la ligature des deux reins.)* When he deemed that the mortification was complete, the ligatures were removed, and the wounds were united by means of stitches: in a short time they healed. The animal, from the day of the operation, became exceedingly low and feeble; but it lived for nearly a fortnight. It was killed by opening the jugular vein, for the purpose of collecting as much of the blood as possible. Not more, however, than a pound could be obtained, before the animal expired.

M. Marchand took 400 grammes of this, and evaporated it to dryness in a sand-bath: rather more than two grammes of urea were thus obtained. Traces also of urea were detected in the fluid, which had been rejected from the stomach by vomiting.

In human pathology we occasionally meet with cases, which exhibit somewhat analogous phenomena, as in ischuria renalis, Bright's disease of the kidneys, the Asiatic cholera, &c. &c. In all of these diseases the quantity secreted, as well as the quality, of the urine is greatly changed from a state of health. The urine voided during an attack of Asiatic cholera has been found to be quite destitute of any traces of urea.

In one or two cases of this disease, M. Marchand succeeded in detecting urea in the blood, which had been drawn during life. It is well known that several physicians have proved the presence of this animal salt in the blood, in some cases of Albuminuria, or Bright's disease of the kidneys.

We have already alluded to the remark of M. Marchand as to the occasional existence of urea in the fluid of dropsical effusion.

M. Nysten was, perhaps, the first to announce this curious pathological fact in 1811; but his memoir, although presented to the Royal Academy of Sciences in that year, was not published till a few years ago. He discovered urea not only in the dropsical fluids, but also in the fluids which were rejected from the stomach, when there was a complete *ischuria renalis.* Hitherto but few experiments have been made on this subject; but it certainly seems highly probable that the conclusions of M. Nysten are quite accurate, when we call to mind the results of the mortification of the kidneys, as induced in M. Marchand's experiments.

We may here allude to the circumstance of saccharine matter being occasionally discoverable in the blood of patients affected with Diabetes mellitus: the

---

* The particulars of this operation are not given by the author, nor the manner in which it was performed. He alludes to the experiments of MM. Muller and Peipers (Archives für Physiologie, 1836,) as having suggested to him the mode of producing the mortification of the kidneys by means of a ligature.

saccharine matter seems to occupy the place of the urea, as it is well known that this latter principle is almost or altogether entirely deficient in the urinary secretion.—*L'Experience.*

---

### On the Action of Diuretic Medicines.

The urinary secretion is liable to numerous changes, in respect both of its quantity and of its properties. In diabetes, the quantity is very greatly increased; whereas in almost all diseases, which are attended with copious perspiration or with diarrhœa, as well as in the various forms of dropsy, the urine is generally scanty. Again; as to the quality of the secretion we usually find that it is alkaline in chronic diseases of the brain and of the spinal marrow, highly charged with uric acid or with urate of ammonia in gout and rheumatism, albuminous in certain dropsies, in structural disease of the kidneys, &c. and saccharine in one form of diabetes.

Various medicines exert a powerful effect on the urine; operating either directly on the kidneys themselves; or indirectly and through the medium of other organs, as the skin or bowels; or lastly by acting on the blood itself and inducing certain changes in its composition. It is well known that many substances, such as various salts, turpentine, and several colouring matters, are discoverable in the urine, in a short time after they have been taken into the system.

But to confine our remarks to the question before us—viz. the action of diuretics, or those medicines which increase the flow of the urine—we may observe that *diuresis* may be produced either by the direct excitement of the kidneys themselves; or by the removal or counter-action of any existing circumstances, which may be impeding the secretion; or lastly by the use of a large quantity of fluid which thus passes through the system and is subsequently discharged partly by the skin and partly by the kidneys.

In considering the action of diuretic medicines, it may be well to divide them primarily into those which are so *in health,* and into those which increase the urine *only in a state of disease.*

The former class or section may be subdivided into

1. *Acrid Diuretics.*—These act directly on the kidneys, and, if used indiscreely, may cause inflammation of their tissue, hæmaturia, and strangury. Cantharides, squill, colchicum, mustard, mezereon, &c. belong to this class.

2. *Exciting Diuretics.*—These act as general stimulants of the system. They quicken the circulation, and thus cause a greater quantity of blood to pass through the kidneys than usual. But independently of this systemic effect, they act, even in small doses which do not affect the general circulation, on the kidneys—as is distinctly observed when these organs are at all inflamed or are otherwise very irritable. Alcohol, æther, the ethereal oils, resins and balsams, are the chief *excitant diuretics.* Turpentine may be considered as intermediate, and forming a link, between the former and the present division of this class of medicines.

3. *Saline Alkaline Diuretics.*—These act by first modifying the state of the blood, and by then stimulating directly the kidneys themselves. They cannot, perhaps, of themselves induce inflammation of these organs, although they may revive it, when it has recently existed. They increase the secretion, without accelerating the circulation; and hence they are rather antiphlogistic, than phlogistic medicines.

The various salts of the alkalis, more especially those in which they are combined with the vegetable acids, are diuretics of this class.

That these substances act *directly* on the kidneys themselves, after being taken into the torrent of the circulation, is proved by the following circumstances.

*a.* They are discoverable by chemical re-agents in the urine. The acid is often changed, but the basis or alkali remains unaffected. As far as we know, none of the other two classes of diuretic medicines can be recognised in the urine; for, be it remembered that the mere circumstance of the peculiar odour of turpentine, and of a few other substances, being present in the urine, shortly after they have been taken into the system, does not necessarily imply their actual presence in the fluid.

*b.* The degree of local action is not in any degree proportionate to the amount of diuresis induced. On the contrary, it rather seems that the less that the kidneys are irritated the greater is the diuretic effect produced.

*c.* A certain interval of time, from one to several hours, passes between their being taken and the manifestation of their effects. Phenomena, dependent upon sympathetic influence, on the other hand, display themselves very rapidly.

In cases of dropsical effusions the absorption of the fluid, and the cure of the malady coincide with the increase of the diuresis induced by means of the diuretics employed. The diseases too, which are attributed to an acrimony of the blood—as for example, various cutaneous eruptions—are frequently much benefited, and even cured, by the use of these medicines alone. Some physiologists have sought to explain the operation of diuretics in the cure of dropsies, by supposing that they have the power of directly fortifying the lymphatic vessels, and of favouring absorption in this way. But there are no good grounds for this conjecture; and we therefore deem it more wise to believe that they act only indirectly, and that it is by diminishing the serous part of the blood, that they stimulate the lymphatics to compensate for the loss.

We shall now allude briefly to the *second division* of diuretics—which comprehend those which have no immediate or direct effect on the kidneys, and act as provocatives of the urine only in a state of disease, by removing or counteracting any unfavorable existing circumstances.

Of these, bloodletting is one of the most important and efficacious in all plethoric or phlogistic states of the system. Low cooling diet has also a decidedly diuretic effect under such circumstances. Purgatives too, especially those which are saline and those which are hydragogue, are powerful promoters of absorption.

On the other hand, when the system is much reduced, and the vital energies are impaired, the class of tonics, including the various vegetable bitters, steel, &c., is a most potent adjuvant in dropsical cases. In short, very much depends on the *cause* of the effusion, and on the existing state of the constitution at the time that the physician is called. Of late years much attention has been paid to the pathology of dropsy; and there is perhaps not one subject in the whole range of medical literature more worthy of accurate investigation.

It is now established beyond all doubt that numerous cases of dropsy depend upon some disease of the heart. Auscultation has done much to reveal such cases, and has thus led to a more scientific and more successful mode of treatment. Where there is overaction of the heart, the repeated application of leeches over its seat, and the use of antimonials, digitalis, refrigerants, &c.*

---

* Perhaps the best formula is such a prescription as the following.

℞. Sodæ carbonat. .. ℥ss.

Potass. nitrat. .. ℈ij.

Sacchari purific. .. ℥ss.

Aquæ distillat. .. ℥xiss.

Vini colchici .. ʒij.

Spir. æther. nitric... ʒij. M.

Three table-spoonfuls to be taken with lemon juice three times daily.

combined with extreme quietude of mind and body, constitute by far the most efficacious medication, not only in relieving the circulation, but also in dissipating any dropsical effusion, which may exist.

Another frequent cause of certain dropsies is a structural disease, more or less confirmed, of some of the abdominal viscera—especially of the liver and spleen. Perhaps no class of diuretics is so useful in such dropsies as that of hydragogue cathartics—such as gamboge, elaterium, &c. in union with the supertartrate of potass.

A third not unfrequent cause of dropsy—and this department of pathological enquiry has, until of late years, been very obscure—is structural change of the kidneys themselves. This variety of the disease is one of the most serious and least curable of all. All acrid and stimulating diuretics are necessarily injurious. The hydragogue cathartics, such as the compound powder of julep, &c., are to be preferred; and great attention should be paid at the same time to the maintaining of a free perspirable state of the skin.

The only other form of dropsy, to which we shall at present allude, is that which is apt to supervene on inflammation of a serous membrane. This is especially frequent in the chest after pleuritis. In the treatment of such cases, the use of mild mercurials, and of large doses of alkalis, such as the carbonate of potass, constitutes on the whole the best practice. The same remark is applicable to dropsy occurring after scarlatina, &c.—*Archives d'Anatomie, &c. par Muller.*

---

### Efficacy of Mercury as an Antiphlogistic Remedy.

M. Delhaye, the author of the following observations, very justly remarks,—

"The wish to explain everything in diseases is one of the greatest errors in every exclusive system of medicine, whether this be the humoral doctrine, the physiological, or the doctrine of solidism.

There are certain occurrences or facts, which every practical man will admit to be true, and which are yet as mysterious and inexplicable to us in the present day, as they were two centuries ago—thus shewing how little progress has been made in the *physiology of disease.*

In spite of all the ingenuity and earnestness of a very able sect of physicians, who have striven to reduce the various forms of morbid action to a few general and primary elements, we suppose that few, if any, of their disciples will be inclined to deny the existence of certain specific diseases and of certain specific remedies.

Take, for the example, syphilis and its (almost) unquestioned antidote, mercury. Can we give any explanation, in the slightest degree satisfactory, of the essential nature either of the disease itself, or of the *modus operandi* of the drug? Certainly *not.*

Again; is not ague an essential and specific form of febrile action? and is not Peruvian bark its antidote, *par excellence?*

All attempts to explain the intrinsic and real nature of these diseases have entirely failed. In short, we believe that there is nothing exclusive in medicine; and for this reason we do not hesitate at once to express our adoption of a rational eclectism, in preference to all the much vaunted doctrines and systems which have been proclaimed for the last 150 years. It seems to us to be a great error, that of supposing that the science of medicine can ever attain to that exactitude and precision, which appertain to those sciences, which have to do with inanimate matter. A chemical result is invariable and uniform—provided the experiments are entirely alike—at all times and in all places. The same is the case with the facts of mechanics and of the other branches of natural philosophy. But this does not hold good in medicine. No two cases even of the same disease are entirely alike; there is always some trait or mark of difference in the vehemence, duration, or succession of the symptoms; and the art of the wise physician is to detect the *physiognomy*, so to speak, of each case, and to deal with it accordingly. Then, too, the influence of the mind and of the feelings on the course of a disease will never be overlooked by the practical man in directing his treatment.

But to proceed to the immediate object of this paper, we shall first mention a few cases, to illustrate the efficacy of mercury in certain forms of ophthalmia.

*Case* 1. *Chronic Scrofulous* (?) *Ophthalmia.*—A young girl, of a nervous irritable constitution, had for some months been suffering from sharp darting pains through both eyes, intolerance of light—so great that she always kept her head bent upon her chest, and the *tarsi* were quite contracted inwards, &c. and these symptoms were attended by loss of appetite and general feverishness. It was a matter of difficulty to ascertain the state of the eyes, in consequence of the spasmodically closed state of the lids. The cornea of the left eye had partially lost its transparency, and presented a deep ulcer on its lower half. The right eye seemed to be only sympathetically affected.

Dr. Delhaye says that, when this case was first submitted to him, he was a most believing proselyte to the doctrines of the physiological school, and that he therefore advised bleeding, leeches, blisters, low diet, &c. The disease however was not at all mitigated by this treatment.

M. Stievenart of Mons, a distinguished oculist, was called into consultation. Agreeing with Dr. Delhaye as to the nature of the disease, he suggested the omission of all depletory and lowering measures, and the use of small doses of calomel and belladonna—a pill, consisting of a fourth of a grain of calomel and a sixth of a grain of powdered belladonna leaves, to be taken every four hours—of an opiate collyrium, and of frictions upon the eyelids with the extract of hyosciamus, thrice daily.

On the third day after the adoption of this treatment, the patient could look at objects without much uneasiness, and by the end of the week both eyes were well, with the exception of the ulcer on the left one.

It is to be observed that a nourishing and somewhat generous diet was administered at the same time, *malgré les symptomes de gastrite*, adds Dr. Delhaye.

*Case* 2. A child, who from her infancy had been subject to repeated attacks of ophthalmia, was seized in her seventh year with scarlatina, which was accompanied with severe thoracic symptoms. Purulent effusion into the right cavity of the chest took place, and required the operation of paracentesis thoracis. While recovering from this dangerous affection, the eyes, more especially the right one, became the seat of a most distressing ophthalmia. There were frequently recurring sharp pains through the orbits, great intolerance of light, &c. Antiphlogistic measures were used for some time, but without any advantage.

Dr. Delhaye, remembering the happy result of the former case, now adopted a similar treatment, although he was in some degree afraid of a mercurial action in a system so debilitated. The calomel and belladonna were given in small doses, the eyes were bathed with a mildly anodyne wash, and a nutritious regimen allowed. The cure was complete by the twelfth day; and it is worthy of remark that the fistula in the side—for this was still open—had nearly cicatrised by the same time.

*Case* 3. A girl, 18 years old, and of a lymphatic habit, had been subject from her childhood to attacks of ophthalmia, which had caused slight opacity of both corneæ. The present attack was a very protracted one, and was attended with much constitutional disturbance. Leeching, blistering, &c. had been tried without effect.

The treatment, recommended by M. Stievenart was therefore adopted, and, by the end of the second week, the ophthalmia had almost completely disappeared.

*Mercurial Inunction in Peritonitis.*

Dr. Delhaye reports three cases to illustrate the efficacy of this mode of treatment. According to him, two were cases of entero-peritonitis occurring within a week after delivery; the third case was one of chronic entero-peritonitis in a woman 60 years of age.

It seems to us unnecessary to give the details, as the reports are rather prolix and vague.

Dr. Delhaye attributes the practice of mercurial inunction on the abdomen in puerperal peritonitis to M. Vandezande of Anvers, and seems to regard it as one of the greatest discoveries of late years in practical medicine. An ounce or more of the strongest mercurial ointment is rubbed in every 24 hours, until a decided effect is induced.

He strongly recommends the same mode of treatment—the inunction of the mercurial ointment on the limbs, &c. so as to induce a decided ptyalism quickly —in cases of chronic inflammation of the meninges of the encephalon and of the spinal marrow.

His memoir closes with the report of several cases of very troublesome *onychia* —wherein a nail had grown into the flesh of the finger, and had caused severe irritation and sometimes ulceration also—quickly relieved by keeping the parts well covered with the strong mercurial ointment; of chronic scrofulous abscesses and sores treated successfully in the same way; and, lastly, of the phlegmasia alba in puerperal women.—*Bulletin Medical Belge.*

---

### Inflammation of the Umbilical Vein in Infants.

(We are not aware that the attention of English pathologists has been drawn to this disease, although it seems to have been noticed by our continental neighbours both in France and Germany for a good many years).

MM. Osiander, Meckel, and Sasse were the first who described phlebitis of the umbilical cord. M. Duplay has recently had an opportunity of examining several cases of it at the Hôpital des Enfans trouvés at Paris; and the following is a brief abstract of his paper in a late number of the *L'Experience.*

In his *first* case, that of an infant which died on the fifth day after birth, purulent matter was found in the umbilical vein from the navel to its entrance into the liver: the small intestines exhibited here and there points of inflammation and ulceration.

In the *second* case—neither the age, nor the symptoms present during life are mentioned—the umbilical vein was found full of pus, and its parietes were somewhat thickened: the umbilical arteries also contained pus. Purulent matter was found in both auditory passages, and likewise under the arachnoid membrane. Both pleuræ were coated with pseudo-membranous pellicles of recent deposit.

*Third Case.* An infant died on the tenth day after birth, having been affected from the fourth day with colicy pains, diarrhœa, vomiting, and meteorism of the abdomen. The peritoneum was found on dissection to be inflamed and partially coated with a membranous deposit, and there was a sero-purulent effusion in the abdominal cavity. The branches of the vena portæ, and especially the umbilical vein, exhibited a preternatural turgescence: this was found to be owing not so much to congestion, as to a thickening of their parietes. The trunk of the umbilical vein was a full line in the thickness of its walls, and its branches were even more remarkably affected. The cavities of all these vessels were coated internally with membranous deposit.

In the *fourth* case, the infant died on the seventh day after birth, after having suffered from severe pain in the bowels, vomiting, icterus, &c.

On dissection, all the morbid changes characteristic of peritonitis, were discovered: the umbilical vein and its branches were thickened, and lined with purulent matter internally.

*Case fifth.*—An infant died on the third day after birth, in consequence of an erysipelatous affection of the body.

The intestines and liver were found to be inflamed, coated with lymph, and also with a puriform exsudation. The umbilical vein, from the navel to its insertion in the liver, was filled with yellow pus.

In the *sixth* case, the infant was affected with symptoms of icterus, purulent ophthalmia, and an erysipelatous affection of the face, having a tendency to gangrene here and there: it died on the tenth day after birth.

Along with certain morbid changes in other parts, the umbilical vein was found to be filled with puriform matter, and to have its parietes considerably thickened.

*General Remarks.*—Our knowledge of the histoiy of phlebitis of the umbilical cord is too imperfect to warrant us in speaking, with any certainty, on any of its characters or features.

As to the *cause* of disease, M. Sasse and others have attributed it to the irritation arising from the ligature of the cord, and from the ungentle attempts, sometimes made, to squeeze the blood out from it.

The *consequences* or *effects* of the lesion seem to be usually peritonitis, icterus, and rapid exhastion of the vital energies.—*L'Experience.*

---

### On Foundling and Bastard Children in France and in other Countries of Europe.

During the course of last year (1837) the Abbé Gaillard published a very interesting and instructive work, entitled "Recherches Administratives, Statistiques et Morales sur les Enfans trouvés, les Enfans Naturels, et les Orphelins, en France, et dans plusieurs autres pays de l'Europe," and which was *couronné* for its merits by the Academic Society of Maçon. The *first* part is taken up with statistical tables of the number of illegitimate births throughout France, and of the proportion which these bear to the entire number of children born. From these it appears that the number has been increasing progressively each year from the revolution of 1789, down to the present time; that the proportion of illegitimate to the total number of births throughout the kingdom may be stated as one to fourteen; that this proportion is much higher in the department of the Seine, than in any other—being nearly as one to three in it—and that it is invariably greater in the manufacturing than in the agricultural districts of the country, and in garrison and sea-port towns than in others. M. Gaillard alludes to the *diminution de la foi religieuse* as one of the most potent causes of this preponderance.

In the *second* section of his work, the author has given an admirable historical sketch of the *fate* of foundling children among different nations, ancient as well as modern. He shews by numerous authorities that in ancient times the legislature did not at all interest itself in the preservation of these helpless beings. In Athens and Rome they were exposed and left to perish; and we know that, in reference even to the children born in wedlock, these so-called civilised states invested the father with the power of putting them to death, if he deemed that he was unable to rear and maintain them. The practice too of inducing miscarriage seems to have been very common among all ranks of society; and we read that women of high family were in the habit of resorting to this infamous means for the sole motive of preserving their youthfulness and beauty.*

M. Gaillard has clearly shewn that the early teachers of Christianity were the first to stigmatize not only this villainy, but likewise that of exposing children, whether legitimate or illegitimate; and that, when this religion became the established creed of the empire, the most severe penalties were enacted against their perpetration.

The institution of foundling hospitals followed, very probably, soon this happy change of things; for we read that there was one in Milan in 787, and one in Rome in 1212. It appears that it was not till the seventeenth century that such an establishment existed in France.

In the *third* section, M. Gaillard examines with great minuteness all the administrative regulations which have been enacted in France, during the last half-century, respecting foundling children: this chapter, although well deserving of attention, we must pass over.

The *fourth* chapter treats exclusively of the mortality among foundling children.

In 1789 the mortality seems to have been enormous; not more than two in a hundred survived four years. This frightful havoc is now considerably diminished; but still much might be done to reduce it lower than it is. Among the chief causes of the mortality, we may mention the necessary exposure of the infants in transporting them, sometimes for considerable distances, to the foundling or registration houses, and also the deprivation to the infant of the natural means of support, its mother's milk.

M. Gaillard glances at some of the regulations and usages in other European countries touching the maintenance of foundling children. He condemns the custom in America, and recently adopted in Great Britain, of imposing all the weight of supporting the child on the unfortunate mother, who is generally "more sinned against than sinning." Some of the German States, with the view of diminishing the charge to the State of maintaining so many illegitimate children, have tried the effect of prohibiting marriages among such persons as are very poor and not able to maintain their offspring; but this practical application of Malthusian doctrines has only led to a more wide-spread corruption of morals among the lower orders. Russia has fallen into the very opposite extreme; for, in the foundling hospitals there, the children are brought up and educated in a much more expensive manner than could possibly have fallen to their lot in their parents' houses. But it may fairly be objected that such a state of things is, in fact, offering a premium or direct encouragement to bastardy.

M. Gaillard has stated some curious particulars respecting the proportional number of illegitimate births at different seasons of the year. As might be expected, it appears from his researches that many more illegitimate conceptions take place in Spring and Summer than in Autumn and Winter.

The institution of Lent, a festival peculiarly devoted to prayer and fasting, was wisely appointed by the Catholic Church 'au moment où le printems va allumer le feu des passions.'

Another particular stated by our author deserves notice here, and, if confirmed by the researches of others, it is certainly a curious one. He says that the relative number of male to female births is less among illegitimate than among

* To say nothing of the *criminality*, we might denounce the *impolicy* of the practice alluded to. A miscarriage tends much more to impair the constitution than a labour. All professional men will admit the truth of this statement.

legitimate conceptions; and that, in some circumstances, the advantage is even in favour of the female sex.*

The concluding part of M. Gaillard's work is occupied with the examination of the various political and moral questions, which relate to the public adoption and maintenance of illegitimate children; how far it is convenient and proper for a state to institute and support establishments for their reception; how, when admitted, they should be treated and educated; in what manner they should be disposed of when they grow up to adolescence; and lastly how far it should be made obligatory on the parents, when they are known, to contribute specially to the maintenance of their offspring. All these subjects are treated with great ability and good feeling by the author, and we can assure our readers that they will be both pleased and instructed by a perusal of his work for themselves. —*Gazette Medicale.*

---

### ON THE DANGER OF BEING BURIED ALIVE, AND OF THE MEANS TO ASCERTAIN DEATH: ACUPUNCTURATION OF THE HEART, &c.

The writer, M. Bourgeois, alludes to several cases of lethargy, extacy, and trance, which have so completely simulated all the phenomena of real death, that the unfortunate victims have been actually carried to their graves, and sometimes even, *horresco referens,* interred before the dreadful mistake has been discovered. In hospitals such an occurrence is especially apt to happen, in consequence of the patients being at once removed to the dead-room, whenever life is supposed to be extinct.

In some parts of Germany, it is the custom to fix a bell-rope in the right hand of each corpse for four and twenty hours preceding burial; and we read of some *Tales of Terror,* of the supposed dead person having, on awaking, rung the bell, and thus saved themselves perhaps the dreadful fate of being buried alive.

M. Bourgeois assures us that when the general exhumation of the *Cimetiere des Innocens,* at Paris, was made during Napoleon's reign—for the purpose of converting the *locale* into a market-place—many of the skeletons were found in such attitudes as plainly indicated that the miserable tenants of the tomb had resuscitated after interment.

It has indeed been conjectured that the change of posture may have been occasioned by the jolting of the coffin, when it is let down into the grave; but we fear that this explanation is not satisfactory. Certainly it will not account for all cases—we allude to those where the skeletons have actually been found fairly out of their coffins, or where too evident signs remain of the interred having been struggling to escape.

The following strange case of lethargy, is related in a recent number of one of the English periodicals. (Query. What Journal is alluded to? *Rev.*)

A medical man suddenly lost all outward consciousness and power of motion, upon hearing of some overwhelming misfortune. It would seem however, that he continued to be quite sensible, within himself, not only of his own existence, but even of the dreadful situation that he was in.

The sense of hearing too still retained some portion of its action; for he could

---

* Whether this statement be correct or not, we do not understand the ground of our author's deduction, "that it affords an additional argument, in favour of the embryological doctrine, that the fœtus in the early stages of intra-uterine life is *neutral* as to sex, and that the rudiments of the genital apparatus become feminine or masculine in consequence of some ulterior influence or direction, which although inexplicable is not the less real."—(*Rev.*)

distinguish around him the cries of his wife and children, the voice of the medical man called to his assistance, and he understood from what was said that he was supposed to be dead.*

After a certain time, *indeterminable pour lui,* he was aware of the preparations for his burial, and of his being put into and conveyed away in a coffin; he heard the clod of earth rattle upon its lid; and it was not until he was in some degree *sealed (scellé)* that he found himself able to shriek and to move about to prevent the fatal mistake.

Some dreadful cases have occurred where the existence of life was not discovered until the knife of the anatomist had been applied to the body. We are informed that such was the manner in which the Abbé Prevost, the author of some well-known romances, was sacrificed!

M. Bourgeois then alludes to the most efficient means that can be used to resuscitate life in suspected cases. He mentions the insufflation of air into the lungs, the withdrawal of blood in some cases and the infusion of it in others, the employment of galvanism or electricity, and lastly acupuncturation of the heart.

This last remedy he regards as one of the most active and efficacious. The operation has been long practised not only with impunity, but often with wonderful success, by the Chinese. Needles may be inserted into and passed through almost any viscus of the body with perfect safety. Even the heart itself may be treated in this manner, and no unpleasant consequences follow.

This has been done in several of the hospitals of Paris, and more especially in the Hôpital de la Pitié, by Professor Beclard: the needles were allowed to remain for several minutes, and were then withdrawn.

Signor Carraro, an Italian physician, has recently made numerous experiments to ascertain the effects of acupuncturing the heart in resuscitating animals asphyxiated by drowning: he reports very favourably of the remedy.—*(Journal Universel des Sciences Medicales, tome* 29.)

M. Bourgeois suggests that the needles might be made the means of transmitting the galvanic or electrical influence through the central organ of the circulation; but he does not speak from experience.—*Revue Medicale.*

---

Poisoning by Arsenic—Exhumation of the Body, and Conviction of the Criminal, three Years after Death.

The following case may be added to those reported by M. Orfila and others, where the assistance of chemistry has most strikingly led to the detection of crime, many months and even years after its perpetration.

A widow woman, by name Lamothe, had, in November 1833, mentioned to several of her acquaintances that a Madame Chevalier had made her universal legatee, and that she hoped she might be soon in possession of her property, as her friend " ne pouvait pas vivre longtemps." It was long after the date of this foolish speech, that Madame Chevalier did in truth die, after a severe attack of colic accompanied with most distressing vomiting.

* M. Bourgeois afterwards alludes to this curious circumstance that the hearing seems to have been the only sense, which remained partially active in several cases of trance. He says, ' asphyxiated and lethargic patients have often declared that, during all the time that they were in a state of insensibility, they were still in some degree aware or conscious of sounds around them. Several facts establish the fact that soldiers, who were in the act of being buried with military honours, have been awakened from their stupor by the report of the muskets over their grave.'

Public rumour, at the time, accused Lamothe of having committed murder; but it does not seem that it ever reached the ear of the public authorities.

Three years afterwards, the house adjoining to that of Lamothe took fire; and the inhabitants of the village, having never forgotten the tale of Madame Chevalier's death, did not hesitate to accuse her (Lamothe) now of wilful arson.

The magistrates, hearing of these reports, determined to investigate the matter minutely. It was ascertained that Lamothe had some arsenic in her possession at the time of Madame Chevalier's death. They ordered the body to be immediately disinterred—three years exactly having intervened since the period of her death.

The soil of the burying-ground being remarkably dry, the body was found not so much decomposed, as might have been expected. It was forthwith sent off to Paris, to be examined, pathologically and chemically, by Drs. Barruel, Henry, and Olivier—three distinguished men of science in the metropolis. On examining the trunk, it was found completely dry, mummyfied, and almost inodorous; the abdominal parietes, although much shrivelled, were entire. On removing these, there seemed to be no vestiges of the viscera, in consequence of the extreme desiccation, which had taken place. On examining however the parts more attentively, the intestines were found to be reduced to the state of mere membranous laminæ, adhering to each other, and to the spine and edges of the pelvis. In the interstices of these membranous *folia*, a brownish coloured pulverulent matter was observed. All the viscera, parenchymatous such as the liver and spleen, as well as the fleshy and membranous as the uterus and intestines, had become blended together, just as now described.

At one spot only, on the upper two lumbar vertebræ, there was a small portion of a brown substance, waxy in consistence, which was probably a portion of the liver. After these short details, it is unnecessary to add that it was not possible to distinguish the abdominal viscera, one from another.

A small fragment of the diaphragm, on the right side, still remained. The only vestige of the lungs was a brown friable substance in irregular nodules; and the heart was converted into a hard black and fragile mass, resting on the vertebræ.

The entire contents of the abdominal cavity—if such it can be called—were now carefully scraped from the bones of the spine and pelvis, and collected together, the earthy granular matter being separated from the foliaceous or membranous substance.

The earthy matter was first examined. A portion was boiled in distilled water, acidulated with a small quantity of pure hydrochloric acid.

On cooling, the liquor was filtered. A small quantity of brown deposit was collected on the blotting-paper, and the filtered liquor was of the same colour.

Through a portion of this latter, diluted with water, a stream of pure sulphuretted hydrogen gas was caused to pass for the space of two hours. This gave rise to a light brownish precipitate, which was carefully collected and tested. It gave no traces of any metallic sulphuret, and was found to contain nothing but minute portions of chloruret of sodium, phosphate of lime, and oxyde of iron—the usual salts of animal substances.

The deposit on the filtering paper was then dried and calcined in a crucible; the residue was then examined, and found to contain only the salts now mentioned, and to give no signs of metallic sulphuret. Hitherto therefore nothing had been detected to lead to any suspicion of poisoning.

The shrivelled viscera were now subjected to a most careful examination. They were first macerated in rectified alcohol for forty-eight hours, and then withdrawn. The spirit, which had acquired a greenish brown colour, was filtered and distilled in a glass retort. The residue was concentrated in a water-bath to the consistence of a soft extract, and then treated with slightly acidulated water and boiled. Ammonia was added to neutralise the acid; and then a stream of sulphuretted hydrogen was passed through the fluid for a length of time. A deep-brown precipitate was formed in consequence. This was carefully collected on filtering paper; and the strained liquor was evaporated to dryness, until a dark brown residue was obtained. On burning a small portion of this on live coals, an empyreumatic animal odour, followed by one resembling garlic, was perceived.

Another portion was then heated strongly with hydrochloric acid, until all the brown matter had disappeared; and the dry product of this treatment, being first neutralized, gave a bright-red precipitate with nitrate of silver, similar to that afforded by the action of this salt on any of the preparations of arsenic.

There were therefore strong suspicions that arsenic would be discovered in the precipitated matter collected on the filter.

It is unnecessary to particularise the experiments, both in the *dry*, and in the *wet* method, which were performed. Suffice it to say that they proved, beyond all doubt, the existence of this poison. The Commissioners sent to the public authorities, along with their report, specimens of—1, arsenic reduced to a metallic state; 2, arseniate of silver; and 3, sulphuret of arsenic floating in water.

The Court of Assizes at once pronounced judgment against the woman Lamothe, and condemned her to perpetual imprisonment. This took place on the 17th of March 1837—three years and a half after the death of Madame Chevalier.

In closing this brief report, it only remains to add that the experiments necessary in such a case, as the preceding, require the most patient and protracted care and delicacy. A week, at least, is not too long for such an investigation.—*Annales d'Hygiene, &c.*

---

### Case of Torticollis, or Wry-neck, treated by Division of the Sterno-Mastoid Muscle.

Dr. Jules Guerin, the author of the following observations, premises them with remarking: Whenever a novel idea is started, it is almost sure to encounter three sorts of opposition—the opposition of the learned, who will not suffer that any thing can be invented or discovered in the present day which has not been known before; the opposition of rivals, who claim to participate in what they have the laudable regret not to have done; and lastly, the opposition of those *esprits retardataires*, who resist all progress or advance whatsoever.

These three sorts of opposition have all been exhibited against those views on the treatment of wry-neck, which I have recently published. It may therefore be useful to report a few cases from my own practice, as well as from that of other surgeons, in order that medical men may judge for themselves of the soundness of my ideas.

*Case* 1. The patient, a youth 19 years of age, had been affected with permanent wry-neck since his infancy. Perhaps even it was a congenital malformation; but this point could not be determined in consequence of the want of any exact report.

When admitted into M. Guerin's Orthopedic Institution, the following was his condition.

"The head is bent or inclined to the left side, its vertical axis forming with the angle of the vertebral column an angle of 145°. The face is turned round toward the right side, so that the left angle of the lower jaw is in a line with the sternum, and the chin with the sternal end of the right clavicle.

On examining the left sterno-mastoid muscle, it is found to be—especially the sterno-mastoid portion—tense, contracted, very hard, and prominent.

The whole left side of the face is more or less deformed or irregular; the left eyelids being drawn in towards the nose, and the tip of the nose and the left half of the lips downwards and to the left side. The cervical column has a very marked leaning to the right side, and the left shoulder is higher than its fellow. The chest too exhibits several irregularities in its symmetry."

*Treatment.*—Various methods of extension having been tried during several months without avail, M. Guerin resolved on performing the section of the contracted muscle, at about eight lines or so above the sternum.

It was performed on the 2nd of December, 1837, in presence of Messrs. Lisfranc, Macloughlin, Bruni of Florence, Thompson of Edinburgh, and others.

A knife, slightly concave on its cutting edge, was passed under the fold of the integuments, which had been pinched up, and between them and the contracted muscle; and being then gently turned round, it (the muscle,) was divided without any difficulty. A distinct cracking noise was heard at the moment, and at the same time also a noisy blowing sound from the air rushing in to fill the vacuum between the divided ends of the muscle. It is well to be aware of the possibility of such a phenomenon, as a surgeon might be apprehensive of a much more serious accident—that of the introduction of air into a divided vein.

A few hours after the operation, M. Guerin, finding that there was still some resistance to the *redressement* of the head, and ascertaining, by the touch, that the sheath of the muscle had not been sufficiently divided, introduced a bistoury again, and made the necessary section.

The extension was not commenced till the second day after the operation.

By the eleventh day the head seemed to be *entierement redressée:* the mechanical treatment was, however, continued for six weeks more. By this time, *tous les elemens du Torticollis avaient disparus.*

*Case* 2. M. Bray, 22 years of age, became affected with wry-neck when an infant, after an attack of convulsions. The head was inclined over to the right side, and the deformity became greater and greater, as the child grew. When admitted into La Pitié Hospital under the care of M. Lisfranc, the following was the condition of this patient.

"The head is so much inclined over to the right side, that its axis forms an angle of 135° with the axis of the body: the face is turned round to the left side, so that its anterior plane cuts that of the trunk at an angle of 45°, and a vertical line from the right commissure of the lips corresponds with the median line of the sternum: the right shoulder is higher by about two inches than the left one.

The right sterno-mastoid muscle is contracted, as hard as a piece of wood, projecting under the skin, and wholly inextensible.

The whole right side of the face is somewhat irregular and deformed."

The operation of dividing the contracted muscle was performed in the amphitheatre of La Pitié Hospital in presence of MM. Lisfranc, Piorry, Pinel, Grandchamp, and many pupils. It was quickly done by inserting the bistoury between the integuments and the muscle, and then gently turning it round, and dividing the latter. A distinct cracking noise was heard at the moment of division.

Immediately after the operation, the head could be *redressée*, and turned round in various directions quite freely. On the third day, the use of the mechanical permanent extension was commenced.

By the end of a month, the *redressement* was complete; and ultimately the free movements of the neck were completely restored.

*Case* 3. This case occurred in a young girl, the contraction having existed for seven or eight years. The operation of dividing the affected muscle was performed by Dupuytren in 1822. It would seem however, from the report of the case, that the deformity, although very much diminished, was not entirely removed.

*Case* 4, is reported by M. Stromeyer in his treatise, Ueber Paralyse der Inspirations Muskeln, 1826, and occurred in a child eight years of age. The affection was of three years' standing. M. Stromeyer, having introduced a bistoury through a fold of the integuments, and then turned it somewhat round, divided both portions, the sternal as well as the clavicular, of the muscle: a distinct cracking noise was heard at the time. The operation was crowned with success.

*Case* 5. Differed considerably from the preceding cases in several particulars. The affection of the muscle seems to have been merely spasmodic, and moreover to have been intermittent and not permanent. Various means having however been ineffectually tried to relieve the disease, M. Stromeyer determined to divide the *sternal* portion of the muscle. The operation was followed by very great relief for some time; but the severe sufferings of the patient having returned from a renewal of the spasmodic contractions of the muscle, the *clavicular* portion also was divided. For some months the patient continued free from her complaint. The spasms of the neck once more however recurred; and now it was discovered that they were seated in the clavicular portion of the trapezius muscle. A third operation was therefore performed for the purpose of cutting the contracted fibres across. From this period the disease was completely cured, and the patient was enabled *frequenter le monde et le spectacle.**

*Case* 6. Occurred in the practice of Mr. Syme of Edinburgh, and is reported in the Edinburgh Medical and Surgical Journal for April, 1833. The disease, wry-neck, had lasted for upwards of a year, and had resisted the repeated application of blisters and the employment of various other remedies. The contracted muscle was therefore divided; and it may be sufficient to state that the cure was speedy and complete.—*Gazette Medicale de Paris.*

---

### Fistula of the Corpus Spongiosum Urethræ treated by Suture.

The patient had been annoyed with a urinary fistula for a long time, when he was admitted by M. Ricord into the Hôpital des Veneriens, to be operated on after the method proposed by M. Dieffenbach of Berlin.

This method consists in simply passing a needle armed with a silk thread or two through the skin—not more deeply—on each side of the fistular opening, and tying the ends of the threads over a small cylinder of sparadrap or of bougie. A catheter should be first introduced into the bladder, and it should be allowed to remain there for a few days, in order to prevent the urine from pressing along the urethra, and interfering with the process of cicatrisation.

On the following day the parts surrounding the sore were swollen and tender;

---

* Before dismissing this case, the report of which, although very brief, sufficiently explains its leading features—we cannot avoid saying that the treatment appears to us to have been unnecessarily *operative.* We are well aware how difficult, nay, sometimes how impossible it is to judge of the propriety of treatment in any particular case, unless we have seen and examined the patient. To resort however to the section of muscles, which may be affected with convulsive contractions, must always be considered a very harsh resource. We particularly recommend to our readers the perusal of a very valuable paper by M. Recamier—a summary of which will be found in the number of this Review for July last—on "The Efficacy of Extension, Shampooing, and Percussion in Muscular Contractions." *Rev.*

and next day the ligature had given way. Although the catheter was retained constantly in the urethra, the edges of the fistula were always moist with urine. A free suppuration from the ulcerated edges was established, a tendency to granulation and to coalescence was perceived, and there was less and less oozing of the urine. M. Ricord was quite gratified with the progress of the case, and expressed his hopes that the cicatrization not only of the skin but of the spongy texture of the urethra was nearly completed.

Unfortunately a few days after this period a fresh attack of inflammation in the part came on; an abscess formed, and the fistula was re-established. M. Ricord attributed this *contre-temps* to the awkward attempts of the patient in trying to introduce the catheter for himself.

(The report of the case ends here).—*La Lançette Française.*

It must always be a difficult thing to induce the healing of urethral fistulæ. The spongy body of the urethra does not readily take on the adhesive inflammation, and the tendency to healing will be much counteracted by the oozing of the urine, in spite of the constant use of the catheter.

---

### CASE OF HYDATIDIC GOITRE, OR HYDROCELE OF THE NECK, SUCCESSFULLY TREATED BY OPERATION.

Jean Peretti, 50 years of age, had perceived some years ago a small tumor on the right side of the larynx: it gradually became larger and larger, until at length it acquired a very considerable size. The compression which it made on the jugular vein had caused an attack of apoplexy; and moreover the deglutition and the speech also were frequently a good deal embarrassed.

The case having been regarded as one of goitre, had been treated with iodine, burnt sponge, &c.; but without success. The continued and rapid enlargement of the swelling at length occasioned a rupture of one of the subcutaneous veins of the neck, and this was followed by an extensive ecchymosis. At this period the patient consulted Signor Petrali. He found that the tumor occupied the entire right side of the neck from the lower jaw to the clavicle. Anteriorly it exhibited a bilobular appearance, and it felt soft and fluctuating under the finger. M. Petrali, after a minute examination, gave it as his opinion that the tumor was a hydrocele of the neck, and he therefore advised the patient to submit to an operation.

An incision was made along the vertical axis of the tumor, by which the skin, cellular substance, and *platysma myoides* were divided. The cyst was thus fairly exposed; its parietes were shining and tendinous-looking. The fluctuation of fluid within being quite distinct, the cyst was freely opened; a large quantity of a yellow, limpid, and inodorous fluid escaped.

The lower or depending part of the cyst was now found to form a cul-de-sac, which extended downwards under the clavicle. A sort of transverse septum or diaphragm, formed by a fold of the parietes, was observed at this part of the sac: the septum being pulsatile, Signor Petrali at first suspected that it might be the subclavian artery partially displaced; but he soon satisfied himself that this was not the case, and he therefore completed the incision of the parietes of the cyst without any interruption.

The hæmorrhage having been arrested, the cavity was filled with lint, and the edges of the wound were then approximated.

On the fourth day after the operation, while dressing the wound, the surgeon observed at its upper and anterior corner a part of another tumor, which, although not fluctuating, he considered to be of the same nature as the one which had been opened. It was in a great measure concealed by the *sterno-mastoid*

muscle, from under which it projected forwards, on a level with the *os hyoides*. It was strongly pulsatile; but the pulsations were evidently communicated by the carotid artery, which lay adjacent to it. To expose the tumor fairly, it was deemed necessary to divide partially the *sterno-mastoid muscle;* it was then found to extend inwards between the carotid artery, the jugular vein, and the pneumogastric nerve. Having pulled the tumor out, Signor P. plunged a bistoury into it, and gave discharge to a considerable quantity of fluid, similar to what had escaped from the former one: he then passed a canula into the sac, and, through this, a probe which was armed with a seton.

Some days after the operation, the opening of the seton was dilated, and a large tent was substituted for the skein of thread. The suppuration was abundant, and the wound and the swelling rapidly contracted.

There still remained a bilobed tumor on the front and left side of the neck: it seemed to consist of two cysts. Signor Petrali attacked the anterior one first, by making an incision upon it from the os hyoides to the sternum. The cyst was situated under the sterno-hyoid muscles. When opened, a quantity of limpid, citron-coloured, fluid escaped. On this being done, both cysts immediately subsided. The body of the thyroid gland seemed to Signor P. to be quite sound. On the following day, the upper lobe of the cyst was found to be re-filled with fluid: a seton was therefore passed through it. The subsequent treatment of the case is thus briefly stated: Dressing as above, continuation of the two setons, the repeated application of caustic. The cysts became gradually obliterated; and ultimately a perfect cure was obtained.

*Remarks.* Since the publication of M. Maunoir's memoir on Hydrocele of the neck, surgeons have trusted generally to the use of setons alone in its treatment. In many cases however these have been found to be inefficient; and then the practice, which was so boldly and so successfully pursued by Signor Petrali, may be adopted.—*Annali Universali di Medicina.*

---

### Lipoma of Large Size in the Groin successfully Extirpated.

A man, 67 years of age, perceived about thirteen years ago, a small indolent and colourless swelling in the left groin. It gradually increased in dimensions, until at length it hung down between his thighs nearly as low as the knees, *comme une enorme queue de mouton de Barbarie.* It was of the shape of a water-sac; its base being large and broad, and its neck narrow and contracted; its surface was lobulated and covered with large veins at different parts; at one point, near its base, was an ulcer which exsuded a quantity of bloody sanious discharge. The generative organs, it would seem, were not involved in the swelling; as it is stated that 'it was moveable in front of them.' The scrotum, inguinal ring, and crural artery being quite independent of it, Signor Malagodi considered the case as one of sarcomatous Lipoma, and he therefore advised its amputation.

He made a free elliptical incision of the integuments over the basis of the tumor, sufficiently low down to save skin enough to make a large flap, by dissecting it upwards. Having very carefully dissected all round the crural arch, so as to avoid any unnecessary injury of the blood-vessels and nerves which were exposed, he cut across the pedicle of the mass with a few strokes of the knife. The wound was of very large extent, but the loss of blood was not very great. The edges were brought together and retained in position by several small hare-lip needles. The progress of the case was most satisfactory, the cicatrisation being complete at the end of the fourth week.

*Remarks.* Pathologists have differed a good deal in attempting to explain the origin of such tumors, as the one noticed in the preceding report. Many have attributed them to an hypertrophy of the adipose tissue of the part, in which they are developed. But this opinion is in some degree invalidated by the consideration, that they are not unfrequently found in parts, where there is normally very little fat. It is therefore more reasonable to regard these growthe as the product of a new formation, just in the same way as tubercles, scirrhus, &c. are; being developed in textures with which they have no analogy.—*Il Raccoglitore Medico.*

---

### SURGICAL REPORT OF THE PALERMO HOSPITAL.

1. Fungus Hæmatodes—2. Urinary calculi in the female mistaken for diseased uterus—3. Sudden death soon after an operation, &c.

### CASE OF FUNGUS HÆMATODES.

The lecture of Dr. Gorgone upon Encephaloid tissue contains a good report of a case of this formidable growth, attacking the lower part of the thigh. The patient was a man between fifty and sixty years of age. The tumor had, as usual, come on without any evident cause, and when Dr. Gorgone saw it, it occupied the whole of the lower half, and part of the middle, of the thigh. It had commenced on the inner, and had extended round to the outer, part of the limb. The real nature of the disease was not suspected at first.

Its hardness and resistance in some parts, especially round its circumference, as well as its elasticity, and the sense of fluctuation (obscure indeed) in other parts, had suggested the idea of its being a chronic abscess.

The skin was not discoloured, nor did pressure cause much pain in the part, although the patient was never altogether exempt from a certain uneasiness in the limb. Frictions with discutient ointments, blisters, and caustics were at first applied to that part of the tumor which was most soft and prominent.

Then three different punctures were made with a trocar: but serum only and blood flowed out; and on introducing a probe into the wound, it was felt to rest on a soft yielding structure.

Dr. Gorgone now suspected the genuine nature of the malady, and proposed amputation of the limb; but the patient refused to submit to it, and he died in the course of a few months. The following faithful description of the appearances of the diseased mass, on dissection, is given in the lecture.

Addressing his pupils, he says, " You see here a quantity of a soft elastic substance, whitish on its surface, arranged in lobules, not unlike, in appearance, to those of the brain, with clefts between these lobules, and these lobules connected together by a cellular tissue, which is soft and in a decayed state. At some points you perceive a soft, pultaceous, nearly fluid substance, of a greenish colour—the usual effect of the softening and incipient putrefaction of parts exposed to the air.

The whole of this encephaloid mass is inclosed in an enveloping sac, which extends from the knee-joint upwards to the middle of the thigh. The adjacent muscles are in several points diseased in structure, being of a dirty yellow colour, and not unlike the liver in consistence. Portions of encephaloid tumor are seen adhering to the *vastus externus*, high up on the outside of the limb. The crural nerve also has become softened, and exhibits a diseased yellow colour at some places. The femoral artery remains unaffected."

2. The following is an interesting case of *vesical calculus*, which had been long mistaken for disease of the uterus.

Donna Carolina Bonomolo, 22 years of age, had been subject for four or five years to pains in the region of the kidneys, to dysuria, feeling of weight and uneasiness in the perineum, &c. The urine was occasionally loaded with an immense quantity of mucus. As she had miscarried twice, her medical attendant supposed that the uterus had become affected with chronic inflammation, and that the urinary complaint was of sympathetic origin. A variety of remedies had been tried ; but all to no effect.

Three years after the commencement of her illness, the patient began to experience sharp pains darting through the bladder, incontinence of urine, (which was now mixed with purulent matter), and very decided symptoms of hectic fever.

It was by mere accident that the true nature of the disease was discovered. A total suppression of the urine coming on, it was necessary to pass the catheter, and, then for the first time, the presence of a large calculus in the bladder was ascertained.

At this period the bladder was exquisitely sensitive, so that the gentlest pressure on the hypogastrium caused severe pain. The patient was indeed in a lamentable condition : what, with the almost constant desire to pass the urine, the pains which these efforts occasioned, and the debility and emaciation which the febrile irritation had induced, considerable apprehensions were entertained of her ultimate recovery. It was quite necessary to use some decided means without delay. After the irritation and local distress were somewhat abated by rest in the horizontal posture, the employment of opiate enemata, and of mild oleaginous aperients, Dr. Gorgone extracted by the cutting operation, as recommended by Dubois, four rough calculi, three of which were as large as chesnuts and the other one was of the size of a strawberry. On the evening of the day of the operation, a sharp attack of fever, accompanied with agonising pain in passing the urine and with convulsions and delirium, supervened. The use of warm baths, of emollient enemata, and of leeches to the hypogastrium relieved the unpleasant symptoms. These alarming attacks recurred six times in the course of the ensuing month, and upwards of sixty small calculi, and a quantity of sabulous deposit, were discharged with the urine. The patient was for a length of time distressed with the incontinence of the urine ; but this most distressing symptom gradually abated (dileguavasi mano mano), as she recovered her general health and strength.

3. *Sudden Death soon after an Operation.*—There are two cases of extirpation of cancerous mammæ detailed in Dr. Gorgone's report. In one of these, the patient (who had been subject for many years to epileptic fits), sank into an apoplectic state immediately after the excision of the tumor, in consequence of fright, it was supposed, from looking at the wound. Some of the attendants thought that air perhaps had entered into some of the divided blood-vessels ; but the phenomena of the case were different from those, which have been described by Dupuytren and others. There was a complete abolition of sensibility and of voluntary power ; the breathing was stertorous, and the pulse was full and quickened. Five ounces of blood were drawn from the feet, and stimulants were applied to the nostrils to excite sneezing. The patient began to groan and to move the limbs about ; slight convulsive twitches were observed to affect the jaw and eyes, and the breathing became less oppressed. But this appearance of amendment was delusive ; for the woman died convulsed 23 hours after the operation. It deserves to be noticed that this patient had been previously affected with attacks somewhat similar to the fatal one. In some of these, she had lain quite insensible for nine or ten hours.

Dr. Gorgone, in reference to the preceding melancholy event, extracts the following passage from Zimmerman's work : "All the passions, when intense, may cause death, or at least induce dangerous diseases. The most experienced physicians agree in admitting that fatal apoplexy may be induced by sudden fright, fear, anger, or any other violent passion or emotion."

It has been remarked by most surgeons that accidents, connected with nervous disturbance, are more frequently observed after operations in those patients, who have smothered and disguised their sufferings at the time, than in those who have given the natural relief to their pain by cries and lamentations. Dr. Gorgone alludes to several cases, where symptoms of trismus, tetanus, or of epilepsy, accompanied, or not, with delirium or stupor, came on very soon after different operations.

The mere introduction of a catheter into the bladder, has been known to induce general convulsions; and our author reports a case, where a fit of apoplexy followed immediately upon the operation of depressing a cataract.

Surgeons ought therefore to be aware of the possibility of such accidents, and that they are much more apt to occur in patients who may have been previously subject to epilepsy, or other diseases of the nervous system.

---

### ON THE APPLICATION OF RAW COTTON TO ERYSIPELATOUS SURFACES.

M. Reynaud, chief surgeon of the French marine, and professor of clinical surgery, has published a long paper in a late number of the Journal des Connoissances Medico-Chirurgicales, on the good effects of applying raw cotton to erysipelatous surfaces. He was led to try it in such cases, from its acknowledged utility in many examples of burns; all the forms of which—from a simple scalding of the surface to a complete adustion of the integuments,—M. Reynaud has for a number of years treated with covering the parts with cotton. In the milder form of the accident, the cotton often soothes almost instantaneously the severe pain, and thus mitigates or checks the febrile excitement which is so apt to ensue; while in the more severe cases, although it does not prevent the suppuration and sphacelation, these processes usually go on more quickly and more favourably under its application. If the remedy is so decidedly useful in burns, we cannot be surprised at its utility in erysipelas. The burning, stinging pain of the disease, we are informed, very speedily abates, the surface becomes moist and perspirable, the swelling and redness diminish, and the skin recovers its healthy pliancy and softness, with little or no subsequent desquamation of its cuticle. The constitutional symptoms of erysipelas being always in a great measure proportionate to the severity of the local distress, they are necessarily much mitigated, and all the functions quickly resume their normal rhythm. M. Reynaud informs us that he has successfully used the cotton medication in all the various forms of erysipelas, idiopathic and traumatic, without regard to the seat or duration of the disease.

The modus operandi of this remedy is, according to him, by promoting a free exhalation from the surface, and by confining the moist and warm atmosphere, thus induced, around the inflamed surface. A steady and uniform temperature is thereby maintained, and the contact of the air and light—two potent stimulants of the skin—is prevented. The cotton application alone is not sufficient however, it must be admitted, in all cases of burns; nor ought it to supersede the use of other local remedies, when these are deemed proper.

When used, it ought to be well carded, and freed from all roughnesses or foreign bodies. The affected part should be enveloped in a moderately thick cushion of it, and a roller should be then passed loosely around, to confine it in contact with the skin.

Fourteen cases are narrated by M. Reynaud, in proof of the efficacy of the cotton application. In four of these the erysipelas affected the face. The constitutional treatment consisted in the use of blood-depletions, of purgatives and refrigerant diuretics. The cotton was applied to the inflamed parts and kept in its place by the night-cap and by handkerchiefs. All the patients recovered. It is proper to observe, that none of these four cases appear to have been severe. In the remaining ten cases, the erysipelas affected the lower extremities.—*Gazette Medicale.*

There can be no objection to the use of cotton, and other such expedients, in the local treatment of erysipelas. For our own part we are inclined to prefer the use of simple tepid fomentations, constantly applied, to any other topical remedies in this disease. The best way of managing then, is to surround the limb with linen, dipped in warm water or poppy decoction, and then to confine this with an envelope of oil-silk. We have thus all the benefit of a constant and gentle vapour-bath around the inflamed parts.—(*Rev.*).

---

### The Pellicle of an Egg, an excellent adhesive Application to Wounds.

The following letter from a M. Cloquet, we find in a late number of the Bulletin Medical Belge.

"To the Editor,

When reading a notice by Dr. Heusner, in Caspar's Wochenscrift, of the employment of the pellicle of an egg as an application to recent wounds, I was reminded of an interesting anecdote, told me by a veteran soldier of Napoleon.

It was at the siege of Saragossa, when the town had been given up to pillage, and the only inhabitants, that remained, were concealed in cellars or haylofts. No quarter was shewn to any one; men, women, and children were butchered without mercy.

The soldier, my informant, was not more compassionate than his comrades. He was, however, moved with pity at the sight of a poor infant, which he found lying under its cradle, and which must have fallen a sacrifice, had he not interposed. He took it up in his arms, and was conveying it away to some place of safety, when he received a sabre-cut across the face, which nearly severed his nose. The flap hung down upon his upper lip, and the wound bled very profusely.

Fortunately a chemist's shop was near; he went in, and the *pharmacien* treated him with the greatest kindness—'Oh! he was no Spaniard,' repeated the old invalid, over and over again, 'for *he* would have poisoned us.'—The wound was cleaned, and the detached flap of his nose was brought together: when the bleeding had ceased, he took an egg and broke it, and then separating the pellicle of the shell, he spread this over the nose (en coiffa le nez du soldat.)

At this time the drums beat to quarters; off, therefore, he had to be on the moment; and from that time to the end of the Spanish campaign, I forgot, said the old man, all about my nose; and at length when I looked at myself in a glass, 'j'etais encore joli garçon.' The scar that still remained shewed that the wound had been a deep and severe one.

Since I was told this story, I have repeatedly made use of the simple remedy alluded to; and, on more occasions than one, I have had to thank the old soldier for the useful hint he gave me."

---

### Diffuse Inflammation of the Scalp, in which a large Extent of the Cranium exfoliated, and the Patient recovered.

Diffuse inflammation of the scalp—unfortunately not an unfiequent attendant upon even slight wounds and injuries—is one of the most serious diseases which the surgeon is called upon to treat. It may prove fatal at different periods of its progress. In some cases, death ensues during the acute stage, in consequence of suppurative meningitis: the patient dies comatose or hemiplegic. On dissection, the dura mater is found detached from the cranium, and bedewed or bathed with pus. The pus is either collected at one spot, or diffused between the meninges. In such cases, pus in large quantity may also be found under the scalp, or between the pericranium and bone.

At other times, the patient sinks, at a late period, from the absorption of purulent matter into the system. The violence of the early stage having passed over, numerous abscesses form under the pericranium, and the pus diffuses itself extensively under this membrane. The openings, either spontaneous or made with the knife, give vent to the pus, and with it are mixed shreds of dead cellular and aponeurotic tissue; and the cranium is found bare at several points. Acute symptoms again make their appearance; the fever of purulent absorption is lighted up; the pus from the wounds becomes vitiated; and the patient dies, with all the symptoms that indicate visceral abscesses.

In a third set of cases, the patient perishes from external hæmorrhage.

Some of the considerable branches of the temporal or occipital arteries become ulcerated, and the loss of blood may prove suddenly fatal.

A case of this sort occurred in the ward of Hôtel Dieu, in 1830.

Lastly, the case is sometimes very tedious, and does not prove fatal for a long period after the subsidence of the acute symptoms.

The outer table of the skull, having been deprived of its nutritious envelope, dies, and Nature attempts to throw off the dead bone; but the strength of the patient generally sinks before this slow process can be effected. Sometimes, indeed, we meet with a fortunate exception to this remark. The following is a rare example of this nature.

A young man was admitted into the Hôtel Dieu, with a small wound on the head. He was seized with diffuse inflammation of the scalp. The acute symptoms having subsided under a most active antiphlogistic treatment (venesection and the use of large doses of tartar-emetic,) suppuration set in: incisions were made at different points to discharge the matter and the sloughing shreds of cellular membrane. The whole of the external table (toute le table externe) of the cranium became necrosed (!) It was necessary from time to time to make numerous incisions to extract the different portions of detached bone. After six months of this 'travail eliminatoire,' the patient was discharged cured.

Dupuytren found that the different sequelæ, when put together, equalled in extent the whole convex surface of the cranium. (!)

The treatment of this formidable affection—diffuse inflammation of the scalp—is well understood in the present day, although the surgeon is often disappointed of success. In general it is necessary to adopt vigorous antiphlogistic measures at first. The use of the tartar-emetic, as recommended by Dessault, ought never to be neglected. But constitutional remedies alone will not do. One or more free incisions through the entire thickness of the scalp, and fairly down to the bone, must be practised.

When suppuration is established, Larrey advises that the wounds be dressed with compresses wetted with camphorated vinegar.—*Bulletin de Therapeutique.*

---

### ENCYSTED TUMORS—SEROUS, ATHEROMATOUS AND MELICERIC—TREATED BY INCISION AND INJECTIONS.

*Case* 1. A young child, who had long been troubled with an impetiginous eruption on the scalp, had a soft movable tumor, as big as a walnut, situated in front of the larynx. It had been as large as a hen's egg, before it was lanced. The contents were a limpid serosity mixed with a little blood. Eight days after the incision, the cyst had refilled, and it was again punctured.

An attack of erysipelas then came on, and, for a fortnight, an oozing of pus took place from the wound. When the erysipelas subsided, all the loose cellular tissue in front of the neck became infiltrated, so as to form ' un poche volumineuse.' Several acupunctures were made; but the sac, in two days after it was emptied, was found to be more filled than ever.

When M. Magistel saw the patient first, the swelling was as large as an ostrich's egg, and extended over all the fore part of the neck, He made an incision of about an inch in length, and discharged two glassfuls of a serous fluid, mixed with flakes of albumen and striæ of blood. For the following two days, an oozing of a similar fluid continued: the lips of the wound were kept apart by pieces of lint. On the third day, M. M. injected some warm 'vin aromatique miellé' into the sac, and kept compresses, wet with the same, on the swelling, by means of a bandage passed round the neck. The sac became somewhat painful and inflamed, and the patient was feverish for the next twenty-four hours. When these symptoms subsided, the injection was repeated daily for about a week. The lips of the wound were touched occasionally with the nitrate of silver. The cure was complete in about a month from the date of M. M.'s first visit.

*Case* 2. A gentleman, advanced in life, had been annoyed for twenty years with a tumor, seemingly a fatty one, situated in front of the neck. It was of the size of a small egg.

During the last six months, it had become much softer, and the skin over it was thin and purplish. M. M. opened it freely by a vertical incision. A grumous cheesy-looking pus was discharged. The parietes of the cyst were of a reddish colour, and exhibited numerous minute projections, like mucous follicles. From these there oozed out a liquid, similar to the contents of the sac. In a day or two, M. M. had recourse to an injection, consisting of decoction of cinchona; and compresses, wet with the same, were applied over the surface of the swelling, the lips of the wound being prevented from closing. This mode of treatment was continued for a fortnight. The wound then healed, and the cure was completed in about a month.

*Case* 3. An eminent physician had, for a number of years, a small wen on the left temple. It began to increase in size and to become soft; and when M. M. saw it, it was as large as a hen's egg. Most of his surgical friends advised him to have it excised; but, professional-like, he did not approve of this summary mode of treatment. He consented, however, that M. M. should lay it freely open. The contents were "un pus melicerique." The injections, which were first used, consisted of "eau miellée," and afterwards of decoction of cinchona. On the fourth day, M. M. extracted with the forceps a tough, whitish, fibrinous membrane, which was no doubt a portion of the cyst. The wound required to be occasionally touched with the nitrate of silver. In three weeks from the time of the operation, the cure was complete.—*Gazette Medicale.*

In reference to these cases, we have only to remark that, when the sac of an encysted tumor can easily be removed, the surgeon should always do so. It is useful, however, to know that the cure may frequently be effected by the free incision of the swelling, and the evacuation of its contents; for, in some instances, the sac adheres with great firmness to the surrounding integuments, and is not readily dissected from them.—*(Rev.)*

CASE OF ILEUS, IN WHICH GASTROTOMY WAS PERFORMED.—DEATH.

Annette Rondot—25 years of age, stout and healthy, with the exception of occasional bilious attacks, which were always accompanied with most severe colic in the epigastrium—was in the month of March last seized, after one of her usual ailments, with acute pain in the right iliac region. This was attended with diarrhœa. Three days subsequently, a large hard swelling made its appearance in the suffering part. For two months the diarrhœa continued, with only occasional remissions, and alternating every now and then with fits of vomiting. The symptoms seemed to be always aggravated soon after eating, and also whenever the bowels were constipated. In the second week of May, she was admitted into the Hospital Cochin at Paris. The swelling, occupying the ileo-cæcal region, was firm and hard, but not very sensitive on pressure. Leeches were applied freely both to the swelling itself and also to the anus, but without any decided benefit. An obstinate constipation now came on; and this was accompanied with vomiting at first of bilious, and subsequently of distinctly fæcal, matters. Fortunately, after all other means had failed, a pill of croton oil succeeded in acting upon the bowels: the symptoms, which had been very alarming, were quickly mitigated, and the swelling considerably subsided.

But the relief was only temporary; for, on the following week, the constipation, fæcal vomiting, hiccup, &c. returned; and these symptoms now resisted the use of croton oil, as well as of every other remedy that could be devised.

The state of the patient became so alarming, that M. Monod, one of the surgeons of the hospital, recommended that the operation of gastrotomy should be immediately performed.

An incision through the integuments and the layers of the abdominal muscles having been made over the tumor, and the peritoneum cautiously divided, a portion of intestine, which proved to be *colon*, protruded: this was immediately replaced by the operator, and, inserting his finger into the wound, he drew down gently a loop of the small intestine, which was red and tumefied, and made an opening into it with scissors to the extent of an inch and a half, or so. A quantity of fæcal matter flowed out, and the patient confessed that she found great relief. A ligature was passed through the mesentery of the divided intestine, and retained at the edge of the wound by means of strips of adhesive plaster: light dressings were applied, and the patient put to bed.

On the following day, the loop of intestine was found to have retracted inwards; but it was easily found, and was then fixed more securely than before, by means of two sutures. The condition of the patient became rapidly worse, and she died on the evening of this day.

*Dissection.* On opening the abdominal cavity, some sero-purulent fluid flowed out; the convolutions of the intestines in the pelvis were coated with semi-concrete pus, and were redder than the other portions of the canal. The intestine, which had been opened in the operation, proved to be the *ileum*, eight or nine inches above the *caput coli*; a very trifling adhesion had taken place at the seat of the artificial anus. On examining the intestinal canal for the purpose of discovering where the obstruction had been seated, it was found to be at the point of junction of the *cæcum* with the ascending *colon*: the contraction of the tube was so considerable, that the point of the little finger could scarcely be passed through it. The *cæcum* rested posteriorly on an indurated mass of scirrhous-like formation; but the mucous coat of the gut was not injured. The other portions of the intestinal canal exhibited no marks of disease, with the exception of patches, here and there, of redness.—*Archives Générales.*

*Remark.*—The propriety of resorting to gastrotomy in such cases as the preceding, appears to us to be more than doubtful: "il vaut mieux abandonner à la Providence des malades aussi desesperés."—*Rev.*

### Case of Chronic Abscess in the Left Iliac Fossa bursting into the Intestine—Cure. Remarks on Pelvic Abscesses.

M. Bricheteau, one of the most enlightened physicians of the French metropolis, has reported an example of pelvic abscess, which recently occurred in his practice at the Hospital Necker.

A young woman, of a scrofulous habit of body, had for a length of time been subject to frequent attacks of excruciating pain in the left lower extremity, from the hip downwards; these were believed to be of a neuralgic character. No means, that had been used, had succeeded in procuring for her more than only temporary and occasional relief. When the case appeared almost hopeless, an unexpected cessation of the sufferings took place, immediately after a diarrhœa, which came on spontaneously. It was now found that a considerable quantity of pus, mixed with blood, was discharged with the alvine evacuations. M. Bricheteau, in consequence of this, began to suspect that an abscess might probably have been formed in the left iliac fossa, where a sense of pain and uneasiness had been felt, and had suddenly burst into the colon or rectum; and thus that the pressure upon the crural nerves, to which the neuralgic pain may have been owing, was removed.

The relief to all the symptoms, which had so long distressed the poor patient, was most gratifying: her sleep and appetite returned, the purulent discharge by the anus gradually ceased, and in the course of a few weeks the patient appeared to have quite recovered.

*Remarks.*—M. Bricheteau is of opinion that there can be little doubt that, the present case was an example of those slow-forming abscesses in the iliac fossæ—most frequently however the right one—which are almost always accompanied with very obscure and perplexing symptoms at first, and the nature of which is often not discovered until they burst into some portion of the lower gut, and their contents are thus discharged *per anum.*

The history of such abscesses was first clearly examined by M. Dupuytren, and an excellent account of his researches was published by the late M. Dance in the Repertoire Anatomique of M. Breschet for 1827. The sixth, seventh, and eighth cases, reported in this memoir, are examples of abscesses in the right iliac fossa, which terminated fortunately after the evacuation of their contents by the bowels. In the fourteenth case, the abscess—apparently the result of difficult parturition—was seated in the left iliac fossa: the purulent matter made a way for itself outwardly through the neck of the uterus. In the fifteenth case also, the abscess was on the left side, and the matter had escaped into the bladder and was discharged with the urine. All these five cases did well.

We have already stated that these pelvic abscesses are formed more frequently in the right than in the left iliac fossa. Dupuytren accounted for this by the circumstance of the situation of the *cæcum* on the *former side,* and the local irritation which may be supposed to be frequently occasioned by the lodgment of hardened fæces in this portion of the intestines. It is also to be noticed that the *cæcum* is not covered by the peritoneum on its posterior surface, but is attached to the pelvis by loose cellular substance. It is in this loose tissue that suppuration always takes place most readily. On the other hand, the sigmoid flexure of the colon is invested with a peritoneal covering all round: hence, when an abscess is formed on the left side of the pelvis, the matter—not making its escape into the bowel so readily—often works its way downwards, and is discharged either under the crural arch, or perhaps at the inguinal aperture.

Before closing these remarks, we may mention that a case, in almost all respects similar to the one reported by M. Bricheteau, occurred lately to M. Berard: It had been long mistaken for an example of idiopathic coxalgia, and its true nature was not discovered, until the discharge of purulent matter took place from the rectum.

In addition to the memoir of M. Dance, already referred to, the *Leçons Orales* of Dupuytren may be consulted with advantage.—*Archives Générales.*

---

### On the Most Frequent Causes of Sudden Death. By M. Devergie.

It has long been, and still is, a prevalent idea, that by far the most frequent cause of sudden death is apoplexy—that form of it to which the French writers apply the term *foudroyante.*

The Statistical Report, made each year to the Prefecture of the Police in Paris, usually attributes four-fifths of the sudden deaths in that city to this disease.

M. Devergie having, for several years, had the medical direction of the Morgue, (an establishment to which the bodies of those who have died suddenly are carried,) has had his attention directed in an especial manner to this important point of pathology; and the following abstract of his researches will, therefore, be read with no inconsiderable interest.

He premises, by remarking that it is only in a few cases that the real or material cause of a sudden death can be predicted from the symptoms, which may have preceded and accompanied the fatal event: and, therefore, that unless a dissection of the body is performed, there must always be a greater or less degree of ambiguity.

The immediate cause of sudden death may be situated—as was first well explained by Bichat—either in the brain, or in the heart, or in the lungs. Perhaps, however, in most cases, two of these vital organs are usually affected at the same time. Thus sudden death, from an affection of the brain alone, is of rare occurrence: the lungs are, in most cases, simultaneously implicated.

Out of forty instances of sudden death, examined by M. Devergie, in four only was the immediate cause, it would seem, of the catastrophe seated in the brain *alone;* in three, the brain and spinal-marrow were congested; and in twelve, the lungs and brain had been affected simultaneously.

Sudden deaths, from disease of the lungs alone, are of more frequent occurrence. Out of the forty cases, there were twelve belonging to this category; and if to these we add the other twelve, in which the brain was affected at the same time with the lungs, we see that in twenty-four out of forty cases of sudden death, the lungs were more or less immediately the seat of the fatal mischief.

Sudden death from cessation of the heart's action, or syncope, is the most rare form of all: M. Devergie met with three cases only out of forty.

The remaining cases—to complete the number of forty—were examples of hæmorrhage, either from the heart, or large blood-vessels, or from the stomach.

From these statements, we may infer that the relative frequency, in respect to the vital organ primarily affected, of sudden death is attributable—

1. To the lungs;
2. To the lungs and brain conjointly;
3. To the brain and spinal marrow conjointly;
4. To the brain alone;
5. To hæmorrhage, whether the blood be discharged outwardly, or be effused into an inward cavity;
6. To the heart.

M. Devergie proceeds to observe that sudden death is very rarely occasioned by local lesion of any of the vital viscera, when this lesion is of very limited extent. Almost always, one of these organs is affected *dans tout son ensemble;* or two, or all, of them are simultaneously implicated.

He regards the current opinion that apoplexy—that is, a circumscribed cerebral hæmorrhage—is the most frequent cause of sudden deaths as entirely erroneous: for in *one* only out of the *forty* cases, all of which he examined with much care, did he discover *un seul foyer apoplectique;* and, even in this case, the lateral ventricles contained a considerable quantity of serum. M. Devergie is of opinion that, in almost all cases of sudden death from cerebral apoplexy, the congestion is not confined to one spot, but is exercised over a considerable extent of the brain. He relates the following case in confirmation of his views.

A woman was seized with the pains of labour; the uterine contractions were soon accompanied with very troublesome fits of vomiting. At the end of four hours she expired. On dissection it was found that a hæmorrhage had taken place from a blood-vessel adjoining to the left lateral ventricle of the brain, and that the blood had gradually filled the four cerebral ventricles to complete distention.

The common idea, that sudden death is often occasioned by a very limited or circumscribed local lesion, has arisen from pathologists being apt to examine the organs separately and by themselves, and not in connexion with each other. Hence the great importance of the anatomist making a general inspection of the cerebral and thoracic viscera, before he divides or detaches any of them from their natural position.

Each of the three modes of sudden death—according as the brain, the lungs, or the heart, has first ceased to act—leaves certain pathological changes, which to the experienced eye are, in some degree at least, characteristic.

Thus, when the death proceeds from some interruption of the pulmonary functions, the circulation is primarily arrested in the lungs:—hence the pulmonary arteries, the right cavities of the heart, and the venæ cavæ are gorged with blood; while the pulmonary veins, the left cavities of the heart, and the aorta are altogether or nearly empty of blood.

Again; if the death has been owing to a sudden cessation of the brain's influence, the meningeal veins will be found extremely congested, the lungs will be similarly affected, and the cavities of the heart on both sides will probably contain blood, although more certainly may exist in the right than in the left cavities. These pathological phenomena are just what we might expect, when we call to mind that, in sudden death from a cerebral cause, the lungs are oppressed before the heart ceases to act.

Lastly; if the death has *debuté* from the heart, the cavities of this organ, on both sides, will be found on dissection to contain blood; the veins are not more loaded than the arteries, and neither the lungs nor the brain exhibit the signs of genuine congestion.

It thus appears that it is quite possible to distinguish, or at least to form a probable conjecture of, the three modes of sudden death, provided we examine the state of the brain, lungs, and heart with sufficient care, and without detaching them from their connexions.

Having thus briefly alluded to the leading pathological characters of sudden death,—whether it proceeds from syncope, or from pulmonary, or from cerebral, asphyxia—we shall now arrange the forty cases, to which we have already alluded, in a tabular view, so as to exhibit more exactly the nature of the disease which proved fatal.

| | |
|---|---|
| Apoplexy, with a circumscribed effusion or *foyer* in the tuber annulare | 1 |
| Apoplexy, meningeal .. .. .. .. .. .. .. .. .. .. .. .. .. | 3 |
| Apoplexy, serous, and accompanied with pulmonary congestion .. .. | 2 |
| Congestion, cerebro-rachidian .. .. .. .. .. .. .. .. .. .. .. | 3 |
| Congestion, pulmonary .. .. .. .. .. .. .. .. .. .. .. .. | 12 |
| Congestion, pulmonary and cerebral .. .. .. .. .. .. .. .. | 12 |
| Hematemesis .. .. .. .. .. .. .. .. .. .. .. .. .. .. | 2 |
| Syncope .. .. .. .. .. .. .. .. .. .. .. .. .. .. .. | 3 |
| Rupture of the heart .. .. .. .. .. .. .. .. .. .. .. .. .. | 1 |
| Rupture of the pulmonary artery .. .. .. .. .. .. .. .. .. .. | 1 |
| | ——40 |

(M. Devergie here enters into a minute anatomical description of the post-mortem phenomena in each of the three sorts of sudden death; but, as we have already alluded to the general pathognomonic characters of these, we deem it unnecessary to do more than recommend our readers to peruse the memoir for themselves.)—*Rev.*

As to the determining causes of sudden death, it is almost impossible to give any satisfactory account of these. One sixth of the bodies, which are brought to the *Morgue* at Paris, is never claimed; and in very many cases of the remaining number no precise information can ever be obtained. In *fourteen* however out of the *forty*, M. Devergie satisfied himself that the sudden death was owing to excessive intoxication; which will account for the frequency of pulmonary and cerebral congestion, as a post-mortem phenomenon in his researches.

With respect to the age of the deceased, this was ascertained in 35 out of the forty cases:—

| | | |
|---|---|---|
| From 20 to 30 | years | 2 cases. |
| From 30 to 40 | .. | 7 .. |
| From 40 to 50 | .. | 10 .. |
| From 50 to 60 | .. | 6 .. |
| From 60 to 70 | .. | 8 .. |
| From 70 to 80 | .. | 2 .. |

As might be expected, the frequency of sudden death among men is very much greater than among women. The ratio in M. Devergie's practice has been about one to eight or nine.

The author sums up his observations in the following propositions:—

1. The most frequent immediate cause of sudden death is pulmonary congestion; either simple, or complicated with cerebral congestion.
2. Sudden death from apoplexy is of much less frequent occurrence than has been generally supposed.
3. In most cases, the sanguineous congestion of any of the three vital organs is usually extensive or diffused, and not confined to a single or very circumscribed spot.
4. Sudden deaths are greatly more frequent among men than among women; and the periods of life, at which they are most common, are between 40 and 50, and between 60 and 70 years of age.
5. Sudden deaths are more common in Winter than in Summer.
6. Intemperance is one of the most common exciting causes.—*Annales d'Hygiene, Juillet,* 1838.

*Remarks.*—The preceding observations of M. Devergie are doubtless quite correct, as far as his own researches have gone; but we strongly suspect that if they were taken as illustrative of the question of sudden death in general, and not only among the lower orders, but also among the higher classes of society—among the latter, be it remembered, the accident is most frequent—many of his conclusions would be found to be erroneous. We are to remember that M. Devergie's data have been derived almost entirely from the examination of the bodies—found in the Seine, and in the streets and suburbs of Paris—at the *Morgue.* It is almost unnecessary to say that by far the greater number of these have been the victims rather of *violent,* than of *sudden,* death. If such be the case, we may at once explain the frequency of pulmonary and cerebral congestion found on dissection, and the rarity of cardiac disease. M. Devergie himself has mentioned intoxication as one of the most common exciting causes of sudden death; and yet we all know that life is very rarely extinguished, in less than the space of several hours, from this cause.

We have already stated our opinion that sudden deaths are of more frequent occurrence among the rich than the poor, among men of educated and sensitive feelings than among the humbler classes of the people. We have little doubt that among the former the heart and brain—more especially the former—are the organs primarily affected in most cases.—*Rev.*

---

### Delirium Tremens, History and Treatment of, with Emetics, by Stoll, in 1778.

The ancient writers have confounded under the general term *phrenzy* various affections, which the moderns have discriminated, and which they have sometimes described as new and unnoticed diseases. Among these we may place that bizarre affection, which was well portrayed by the English physician Sutton under the name of *delirium tremens,* and which, he shewed, was in almost every case occasioned by the abuse of spirituous liquors.

In lately perusing the works of Stoll, I (Professor Forget of Strasburg) was much struck with the very exact and faithful delineation of this disease, in the Chapter on the Causes and Seat of Phrenzy.

The report of the following three cases will interest every medical reader, not only by the accuracy of the description, but also by the judicious therapeutical instructions recommended by the author.

*Case* 1. A middle-aged man, of a robust constitution, was admitted into the hospital at Vienna on the 13th of June 1778. For six days he had been distressed with extreme lassitude, loss of appetite, frequently-recurring chills, and subsequently with a great trembling or shaking of the body, as if he was in the cold stage of an ague. The pulse was but little affected ; the speech was quite clear, and the mind was tranquil. Dr. Stoll regarded the case as one of incipient bilious fever, and prescribed some light aperient to act upon the bowels.

During the night, however, the patient was unexpectedly seized with furious delirium ; and, from this time until the hour of the morning visit, he was in a constant state of violent excitement, screaming out, and tearing every thing that he could get hold of. The pulse at this time was vibratory ; and the surface of the body was covered with a copious sweat.

Three grains of emetic tartar were administered immediately ; but neither vomiting nor purging was induced. An hour afterwards a mixture, containing eight grains of the same salt, was prescribed : one half to be taken at a dose, and the other half in half an hour, if no effect was produced. The patient however, mistaking the medicine for wine, which he was continually desiring to have, swallowed nearly the whole of the mixture at once. He was vomited and purged briskly three times ; and a copious perspiration broke out. The delirium soon subsided, and he fell asleep muttering to himself. On the following day, he awoke quite conscious, and almost entirely tranquillized in his mind.

*Remark.* The treatment adopted by Stoll in the preceding case deserves especial notice, as some late writers have claimed to themselves the credit of having first announced the efficacy of emetics in delirium tremens.

*Case* 2. In the Autumn of 1778, we received into the hospital a man-servant, who was at the time in a state of furious excitement, Four or five days previously he had drunk a large quantity of strong beer, while heated from running ; and he was soon afterwards seized with intense headache, and frequently-returning chills. He was immediately bled from the arm ; but, although the blood was buffy, *no decided relief was procured.* During the course of the evening, he fell into a state of delirium : his eyes seemed to be pushed forwards, and they were constantly rolling about ; he screamed out in violent paroxysms of rage ; the whole surface of the body was drenched in profuse perspiration ; and the pulse, though scarcely at all accelerated, was vibratory and jerking. A second venæsection was practised, *but without benefit.*

On the following day, however, he was much more composed, and then Stoll saw him for the first time. He advised a small quantity of blood to be taken again from the arm : it proved to be not at all buffy now. Acidulated drinks and gentle aperients were also prescribed ; and, along with these medicines, a four-ounce mixture containing six grains of tartrate of antimony. These means produced three copious stools, and also vomited him freely thrice. He became, soon afterwards, very calm, and at length fell into a profound soothing sleep, which lasted for many hours. In the course of a few days he had completely recovered.

*Remark.* The inutility of sanguineous depletions was strongly manifested in the preceding case.—*(Rev.)*

*Case* 3. A young man, who had been long addicted to excess in drinking, was seized with shivering, severe headache, frequent vomiting, and general weakness. At times, all his limbs trembled or shook, as if he was in an ague-fit. The pulse was quickened, hard, and full. He was bled and purged with cooling aperients—the blood was buffy. During the course of the night, however, he fell into a state of delirium and excessive restlessness. On the following day, there were frequent attacks of convulsions; and the delirium was almost constant. Next day he was conveyed to the hospital : he was then more tranquil, and could give some account of his illness.

While engaged however in speaking, his looks and whole demeanour indicated an excited and wavering state of the mind. He had no sooner finished his tale, than he was seized with furious delirium.

Five grains of the tartrate of antimony were given at once, and smaller doses were given at short intervals. After the emetic and purgative action of the medicine was freely induced, the state of the patient became much more composed. Although sleep did not come on for some time, his health gradually improved and was ultimately quite restored.

*General Remarks.* These three cases, reported by a most experienced physician of the last century, before the term *delirium tremens* had been heard of, will be read with interest and advantage. The efficacy of the antimonial treatment is most satisfactorily proved by the result of each case ; and the recommendations of it by some recent writers are thus very beautifully confirmed. We are induced to append the following case, as it was one of extreme severity. It occurred in Dr. Forget's practice.

A healthy robust man, who confessed his propensity to intemperance, was admitted into the Hospital at Strasbourg on the 24th of June, 1837. His whole frame was agitated and shaking ; he complained of acute headache, and general restlessness ; the pulse was normal, the heat of the surface moderate, the bowels regular, and his ideas seemed to be clear and but little disturbed. He was bled from the arm ; but the coagulum was soft and not at all buffy. As the patient said that, some years ago, he had had an attack of ague, the present ailment was regarded in the same light; and accordingly quinine was administered. During the course of the night, however, he became vehemently excited, and at length quite ferocious, so as to require the application of a straight jacket.

Next day he was in a constant state of delirium, screaming and shouting out, his whole frame convulsed with a trembling agitation ; the eyes sparkled and rolled about incessantly; every now and then, he made violent efforts to disengage himself from the jacket : the pulse however was not much excited, and the skin was moist.

During the visit, he was seized with a most severe epileptiform convulsion; the whole body became stiff and immoveable, the head was thrown back, the face livid, the jaws and fingers clenched, and the breathing convulsive. This paroxysm lasted for two or three minutes. Twenty leeches were applied to the temples, and one grain of the extract of opium was ordered to be given every two hours: mustard poultices were applied to the legs. The agitation continued more or less during the whole day: the opiate treatment however was steadily persevered in; and by midnight, when he had already taken nine grains in all, he fell into a deep sleep.

Next day, he was calm, spoke rationally, and had lost all the trembling agitation of his body and limbs—the pulse was eighty, and rather small in volume. This day he took three grains of opium, and had an aperient enema. The quantity of opium was gradually diminished each day; and by the end of the week he was discharged quite recovered.

*Remarks.* This case is especially interesting in a diagnostic point of view; its characters having successively assumed those of intermittent fever, meningitis, and epilepsy. It will often require great tact to avoid such mistakes; and, on the correctness of a right discrimination, will depend the appropriate management of the disease. The reader will observe *that the paroxysms of delirium tremens are almost always aggravated after venæsection;* whereas they are often cut short, almost immediately, by a full dose of opium. The delirium, which not unfrequently supervenes after a severe accident, such as fractures of the limbs, &c. (this is the *delire traumatique* of Dupuytren, and by him was always treated with opium), is of the same nature essentially as idiopathic *delirium tremens*, and requires the same mode of medication.—*Bulletin General de Therapeutique.*

---

### On the State of Vaccination in various Countries of Europe; Propriety of Re-Vaccination, &c.

The medical reader is well aware that, within the last few years, great attention has been paid in some of the German States to the question, how far vaccination has proved a preservative against the contagion of small-pox. It has been made an object of government enquiry; and the statistical reports, which have been derived not only from military, but also from many civil physicians, now constitute a mass of most valuable documents.

From these it most unquestionably appears, that the small-pox has of late years been progressively on the increase. According to Dr. Heim, of Wurtemburg, the author of an important work on the subject, nearly 180 out of every 1,000, who have been vaccinated, have suffered from variolous or varioloid contagion.

The results of re-vaccination also may be appealed to in confirmation of the same fact—the diminution of the protecting influence of the cow-pox.

Out of 44,000 cases of re-vaccination, 20,000 exhibited the *genuine*, and 9,000 the *modified* cow-pox vesicle; and in the remaining 15,000 no effects were produced by the experiment.

The state of the cicatrices on the arms of the re-vaccinated has been examined with particular care. Out of 1,322 persons, who presented normal cicatrices, the re-vaccination *took* in about 65 per cent.; in 25 per cent. it exhibited the modified vesicle; and in 9 per cent. it failed altogether.

Out of 1,134 re-vaccinated persons, in whom the cicatrices on the arms were incomplete, 18 per cent. exhibited the modified, and all the rest the genuine cow-pox.*

At the present time, among the German medical men, little or no value is attached to the state of the cicatrices on the arm, as an index of the susceptibility of the system to be affected with re-vaccination. *That this opinion has been the result of observation may be fairly inferred from the circumstance that the early ordonnances, issued by the Prussian government, prescribed the operation to be performed only on such of the soldiers as did not exhibit cicatrices having the characters indicated by Dr. Gregory of London.* In consequence however, of the extension of variolous and varioloid disease among the army, the medical officers have unhesitatingly recommended the universal practice of re-vaccination on all the soldiers, without exception.

In conclusion, we may state that the German physicians are of opinion that the vaccine virus has not in reality lost any of its original powers or become deteriorated, by having been transmitted through so many individuals; but rather that its preservative quality against variolous disease is only temporary, and therefore that vaccination ought always to be repeated after the lapse of 10 or of 14 years.†

The increase of variolous and varioloid disease of late years they attribute not to the *decay* or deterioration of the cow-pox virus, but to the cause we have now mentioned—its protecting power seeming to be limited to a certain time.

Such are the prevailing doctrines of the German physicians; and as the experiments, upon which they are founded, have been made upon a very large scale and under the sanction of the government, they are certainly entitled to great respect.

(We had intended to have described shortly the present state of medical opinion on vaccination, and the questions connected with it, in some other countries of Europe; but this we must reserve to the ensuing number, as the Printer has *ordonné* that—

"Here is our journey's end, here is our butt,
And very sea-mark of our utmost sail."—*Rev.*)

---

* These calculations will be found to vary exceedingly in different reports: they are not therefore to be taken as affording any general conclusion. Thus, in one of the reports from the Prussian army, out of 7845 re-vaccinated soldiers, in all of whom the cicatrices on the arm were distinct and normal, 31 per cent. exhibited the genuine, and 29 per cent. the modified cow-pox; whereas in the remaining 40 per cent. the operation failed in producing any effect. The state of the cicatrices cannot perhaps be at all depended upon, as affording any means of judging as to the liability of the parties to be affected either with the vaccine, or with the variolous, contagion.

† According to this view, it is not necessary to have recourse to the cow for supplies of fresh lymph. There cannot however be any objection to this; and, as the subject is certainly not determined, it might be wise to renew the virus occasionally—especially as we are told that, during the last five years in Wurtemberg, the pock is known to have appeared on 188 cows *dans toute sa perfection.* —*Rev.*

